# Stakeholders’ perceptions of the nutrition and dietetics needs and the requisite professional competencies in Uganda: a cross-sectional mixed methods study

**DOI:** 10.1186/s12913-021-06090-3

**Published:** 2021-01-27

**Authors:** Peterson Kato Kikomeko, Sophie Ochola, Archileo N. Kaaya, Irene Ogada, Tracy Lukiya Birungi, Peace Nakitto

**Affiliations:** 1 Department of Food, Nutrition, and Dietetics, Kenyatta University, P.O Box 43844, Nairobi, Kenya; 2 Department of Human Nutrition and Home Economics, Kyambogo University, P.O Box, 1 Kyambogo, Kampala, Uganda; 3 School of Food Technology, Nutrition and Bioengineering, Makerere University, P.O Box 7062, Kampala, Uganda; 4 Department of Human Nutrition, St. Francis Xavier University, Antigonish, Canada; 5 Uganda Martyrs University, Rubaga Campus, Kampala, Uganda; 6 Elizabeth Glaser Pediatric AIDS Foundation, Mbarara, Uganda; 7 Self Help Africa, Kampala, Uganda

**Keywords:** Competency-based education, Human nutrition and dietetics, Knowledge, Skills, Community, Health systems, Uganda

## Abstract

**Background:**

Effective implementation of nutrition and dietetics interventions necessitates professionals in these fields to possess the requisite competencies for health systems performance. This study explored the stakeholders’ perceptions of the community nutrition and dietetics needs, the nature of work done by graduates of the Bachelor’s degree in Human Nutrition/Human Nutrition and Dietetics (HN/HND), and the competencies required of Nutrition and Dietetics professionals in Uganda.

**Methods:**

A cross-sectional mixed methods design was used. Respondents included 132 graduates of the Bachelor’s degrees in HN/HND obtained from the Makerere and Kyambogo Universities in 2005–2016; 14 academic staff that train HN/HND in the two universities; and 11 HN/HND work/internship supervisors. Data from the graduates was collected through an email-based survey; data from other participants was through face to face interviews using researcher administered questionnaires.

**Results:**

Most HN/HND respondents (84.8%) obtained their Bachelor’s degrees from Kyambogo University; 61.4% graduated in 2013–2016. Most (64.3%) academic staff respondents were females and the majority (57.1%) had doctorate training. All stakeholders viewed communities as facing a variety of nutrition and dietetics challenges cutting across different Sustainable Development Goals. The nutrition and dietetics interventions requested for, provided, and considered a priority for communities were both nutrition-specific and nutrition-sensitive. Work done by HN/HND graduates encompassed seven main competency domains; the dominant being organizational leadership and management; management of nutrition-related disease conditions; nutrition and health promotion; research; and advocacy, communication, and awareness creation.

**Conclusions:**

This study shows that nutrition and dietetics challenges in Uganda are multiple and multifaceted; HN/HND graduates are employed in different sectors, provide nutrition-specific and sensitive services in a multisectoral environment, and are expected to possess a variety of knowledge and skills. However, graduates have knowledge and skills gaps in some of the areas they are expected to exhibit competency. We recommend using these findings as a basis for obtaining stakeholder consensus on the key competencies that should be exhibited by all HN/HND graduates in Uganda; developing a HN/HND competency-based education model and a national HN/HND training and practice standard; and undertaking further research to understand the quality and relevancy of HN/HND curricula to Uganda’s job market requirements.

**Supplementary Information:**

The online version contains supplementary material available at 10.1186/s12913-021-06090-3.

## Background

Malnutrition in its different forms: undernutrition; micronutrient deficiencies; and overweight, obesity, and the associated diet-related non-communicable disease remains a key global health challenge [1, 2] partly due to the multifaceted nature of its causes and the related effects. Despite the widespread recognition that nutrition drives economic growth, social change, and human development [3–6], the United Nations indicated a rise in the number and proportion of undernourished people globally from 777 million (10.6%) in 2015 to 815 million (11.0%) in 2016 [7]. Recent estimates indicate the prevalence of undernourishment to have risen to 22.8% in Sub-Saharan Africa [8] and stunting, in particular, has been identified as one of the key barriers preventing African children from attaining their full growth potential [3]. The limited progress in addressing nutrition at the global, continental, and national levels may slow progress towards the attainment of nutrition targets as set in the Sustainable Development Goals (SDGs), the United Nations Decade of Action on Nutrition, and the 2025 Global nutrition targets [6, 8–10]. Nutrition interventions have been differentiated into nutrition-specific and nutrition-sensitive [11] and recommendations made that their implementation is undertaken at multiple levels from the topmost government to local; engage an interplay of multiple stakeholders under the leadership of Governments, with the support of other actors including the civil society, United Nations, academia, donors, and business; and multiple sectors including those from health, education, agriculture, gender, and other sectors [12]. Nevertheless, multisectoral collaborations remain underutilized in middle and low-income countries [13]. In Uganda, addressing malnutrition is high on the national health and social-economic development agenda [14–18].

Nutrition and Dietetics professionals are a key human resource for the delivery of nutrition-related services [19]; and should thus possess the requisite competencies, i.e. the nutrition and dietetics knowledge, skills, and abilities necessary to perform in the given national health systems settings. To equip health professionals with the requisite performance competencies necessary for meeting the health systems challenges of the twenty-first century, countries have been called upon to undertake reforms in their national education systems by adopting competency-based education (CBE) and inter-professional training and development [20]. “A competency-based approach is a disciplined approach to specify the health problems to be addressed, identify the requisite competencies required of graduates for health-system performance, tailor the curriculum to achieve competencies, and assess achievements and shortfalls” [20]. The mastery of professional competencies is important in fostering the impact and sustainability of individual careers [21]. DiMaria-Ghalili et al. [22] found it critical that the nutrition and dietetics health systems performance competencies needed by each type of health professional cadres are identified and evaluated. Palermo, Hughes, and Mccall [23] in particular, support the need to delineate and differentiate the competencies required for the practice of nutrition and dietetics. Presently, emerging consensus on the need to adopt CBE in health professionals training exists, although more in the developed than in developing countries [24]. In Africa, the Continental Education Strategy for Africa 2016–2025 is focussed on fostering competency-based learning [25].

The government of Uganda embraced the multisectoral approach for the delivery of nutrition interventions in 2011 upon the adoption of a National Nutrition Action Plan and has since called upon all concerned ministries to integrate nutrition in their sector policies and plans [14, 15]. Some of the lessons learnt in regards to multisectoral nutrition policy integration in Uganda point to the need for “training nutritionists and seconding them to occupy strategic positions across sectors” [26]. It is thus vital that the Nutrition and Dietetics professions trained in Uganda get equipped with the requisite knowledge and skills for health systems performance-in a multisectoral context. To date, however, limited research has been undertaken to understand the knowledge and skills required of Nutrition and Dietetics professionals to operate in Uganda’s multisectoral nutrition implementation context. In our recently published paper, we realised that efforts to develop and adopt CBE in Uganda had mainly been demonstrated in medical training and consequently recommended the need to identify and evaluate the competencies required of Human Nutrition/Human Nutrition and Dietetics (HN/HND)^1^ graduates as well as adopt CBE in the training of HN/HND in Uganda [19]. To achieve this, an understanding of the stakeholders’ perceptions is vital [27]. According to Gruppen, Mangrulkar, and Kolars [28], “needs assessments that reflect available health data, input from the community and the public health perspective” can provide valuable information based upon which stakeholder perceptions can be evaluated. Stakeholder evaluations regarding the adequacy of training received in relation to the requirements for practice can be undertaken through surveys that give responsive feedback from graduates, their trainers, and employers [28].

The purpose of this study was to explore the stakeholders’ perceptions of the community nutrition and dietetics needs, the nature of work done by HN/HND graduates, and the competencies required of Nutrition and Dietetics professionals in Uganda. The key questions answered by this study are: (1) What are the nutrition and dietetics related challenges facing different communities in Uganda?; (2) What nutrition and dietetic services are demanded by Ugandan communities?; (3) Which of the demanded nutrition and dietetic services are provided by the different agencies?; (4) What is the nature of work done by HN/HND graduates in Uganda?, (5) and What knowledge and skills are required of HN/HND professionals to perform effectively in Uganda’s varied healthy system’s settings?

## Methods

### Study design and setting

This study used a cross-sectional mixed methods design. The rationale for using this design was based on the need to triangulate data collection and analysis processes as well as obtain complementary data on the study objectives to strengthen the study conclusion [29, 30]. The study population comprised graduates of the Bachelor’s degree in HN/HND obtained from the Makerere and Kyambogo Universities of Uganda in the years 2005–2016^2^; academic staff that train HN/HND in the two Universities; and HN/HND work/internship supervisors from selected regional referral hospitals and Non-governmental organizations (NGOs). Graduates and academic staff of Kyambogo and Makerere Universities were the only ones included because these were the only institutions that had graduated HN/HND professionals in the study reference period. Qualitative description techniques [31] were in particular applied in sampling and data collection. Using a qualitative descriptive approach allows for the collection of data directly from the persons believed to directly experience the matters under study [32]. Data from HN/HND graduates were collected through a cross-sectional email-based survey while that from the academic staff and HN/HND work/internship supervisors was undertaken through face-to-face interviews using a structured key informant interview guide.

Graduates who responded to the survey were not re-interviewed amongst the other categories of respondents. Data collection and analysis were done following an explanatory sequential design [29, 30]; beginning with that from the HN/HND graduates and finally to that for the academic staff and work/internship supervisors. The major point of interface for the quantitative and qualitative methods was in data analysis. The whole process for designing the sampling framework, developing the email-based survey tool, and undertaking data collection from the graduates lasted from December 2017 to August 2018. Data collection from academic staff and work/internship supervisors was undertaken from February 2019 to May 2019 after a preliminary analysis of the data obtained from the HN/HND graduates. Data collection from the graduates took an unusually longer than expected period despite repeated reminders. This was partly attributed to the techniques used in data collection; poor internet connectivity; limited access to computers; length of the questionnaire and the types of study questions; and the general perception by some graduates that study questions were targeted to only the employed graduates. Also, most questions were open-ended and required an appreciable level of personal professional reflection before being answered. A results validation workshop was undertaken in October 2019 to further obtain study participants’ feedback and general insights on the study findings.

### Participant selection

#### Graduates of the Bachelor’s degree in HN/HND

A sampling frame for the graduates was developed from the graduation records of Kyambogo and Makerere University for the years 2005–2016. According to the available records, there were about 607 graduates for the reference period; 493 from Kyambogo University and 114 from Makerere University. The available email and phone contacts for the graduates were obtained from the respective university department records. Starting with these, other contacts were obtained by making phone calls to the known contacts, explaining to them the purpose of the research, the methods that were to be used for data collection and requesting them to provide the email and phone contacts of their fellow graduates. This process was repeated until no more contacts could be obtained; at which point 450 email and mobile phone contacts of the 607 graduates had been obtained. Given that the participants were widely geographically distributed and given the techniques that were to be used in reaching the participants, a decision was made to have an exhaustive sample of all the 450 graduates whose contacts had been established as the study sample and to invite all to participate in the study. The use of such a strategy is theoretically justifiable and meets the requirements for sampling when a study uses aspects of a qualitative descriptive approach in sampling [33] as was in this study.

To collect data from the participants, a Google email-based group account comprised of emails identified while developing the sampling frame was created. A cross-sectional email-based survey was then undertaken amongst all the 450 contacts by emailing participants a consent form (supplemental file 1), a structured questionnaire in a Microsoft word format (supplemental file 2), and a URL link to the web-based version of the questionnaire hosted in Google forms. The options allowed participants to either download the questionnaire sent as an attachment, work offline, and send it back to the researcher, or to directly fill the questionnaire online and submit filled. An acknowledgment receipt was sent for each received response. Email questionnaires were used given their cost-effectiveness in reaching a widely distributed population sample and ease of transmission. To improve response rate; several recommended strategies were undertaken including; providing respondents an extended time to respond, sending out repeated reminders, giving assurance that the responses were vital and would be used, giving assurance of anonymity of responses as these would be de-identified, and providing notification for receipt of responses [34].

#### Academic staffs that train HN/HND

Names of the full-time academic staff that train undergraduates of the Bachelor’s degree in HN/HND in Makerere and Kyambogo Universities were obtained from the concerned university departments. From the lists, the names of seven academic staff who responded to the HN/HND graduates study instrument were identified and excluded. The remaining list had 14 contacts; since the staffs teach different study units, a deliberate decision was undertaken to purposively reach all the 14 identified staff and request for their participation in the study. Data collection was done through face to face interviews using the researcher administered questionnaires (supplemental file 3) and responses concurrently written in a notebook during the interview. Audio recording was also done only for those participants who accepted to be recorded. The interviews were conducted by the principal researcher, in a convenient office at the interviewee’s workplace, on a date and time agreed upon before the actual interview and upon obtaining approved consent (supplemental file 5). On average, interviews lasted for 45 min to one hour.

#### HN/HND work/internship supervisors

The majority of the graduates that responded to the study indicated having undertaken part of their internship training from the national and regional referral hospitals and in several NGOs in different regions of the country. We purposively selected and undertook face to face interviews using researcher administered questionnaires (supplemental file 4) on 11 supervisors of HN/HND interns/employees. Seven of these were based in hospitals (one from the national referral hospital, and six from regional referral hospitals), while four worked in the NGO sector (one worked in a humanitarian agency that was employing most of the graduates that responded to the study, one worked in an HIV/AIDS research-based institution, one worked for an agency whose work was mainly in nutrition and livelihoods promotion, while the other was a director for an NGO mainly dealing in nutrition and health promotion). All interviews were undertaken by the principal researcher, in a convenient office at the interviewee’s workplace, on a date and time agreed upon and upon obtaining approved consent (supplemental file 5). Notes of participant explanations were taken in the course of the interview. Audio recording was also done only for those participants who accepted to be recorded. Interviews lasted for about 45 min to one hour on average.

### Study instruments

In all cases, the questionnaires that were used comprised mainly of open-ended questions and captured aspects of respondents’ demographics; training and employment history of the HN/HND Bachelor’s degree graduates; and general stakeholder perceptions in regards to the nutrition and dietetic challenges faced by community members; nutrition and dietetic services demanded by communities, those provided, and those considered a priority; the knowledge and skills required of graduates of the Bachelor’s degree in HN/HND to competently perform in Uganda’s health system; knowledge and skills gaps amongst HN/HND professionals; the knowledge and skills inadequately attained during training; and stakeholder awareness of the nutrition and dietetics minimum training standards in Uganda.

All study instruments were developed by the principal researcher with support and input from the study supervisors; additional revisions were done based on comments received during the ethical review process. Pretesting of all study instruments was done to further ascertain the comprehension, relevancy of the questions, as well as the general applicability of the study instruments. A participatory pre-test [35] of both variants (Microsoft word and web-based versions) of the HN/HND study instrument was undertaken among two purposively selected HN/HND graduates; one from Kyambogo University and one from Makerere University. The suggestions received were used to improve the questions, ensure that the right options for question types were programmed in the web-based version of the questionnaire, and ascertaining the ease of use of the questionnaire when responding on a smartphone and a personal computer. Undeclared pre-tests [35] were then undertaken on each variant of the HN/HND study instrument using three purposively selected HN/HND graduates for each variant of the questionnaire but no major modifications were found necessary to be done on either variant of the questionnaire. The responses obtained from the undeclared pre-tests were jointly analysed with other graduate responses.

The study instrument for the academic staff was subjected to a participatory pre-test by three academic staff members that train HND in Kyambogo University and revised based on the comments received. Relatedly, participatory pretesting of the study instrument for the work/internship supervisors was also done by two purposely selected graduates, one working in the Humanitarian sector and another working in a hospital setting. The members engaged in the pretesting of study instruments for the academic staff and work/internship supervisors were not interviewed under the two respective categories having initially responded as former HN/HND graduates.

### Researcher standpoint

The principal researcher wore different “hats” as a graduate of HND, a lecturer of nutrition and dietetics at Kyambogo University, and a graduate student undertaking doctoral research. He thus simultaneously adopted both the insider and outsider positions [36] while undertaking this research. Adopting both positions was invaluable in that it enabled the researcher to seek information in a field he had knowledge and experience in, could easily comprehend, as well as triangulate the data from different participant categories. By simultaneously adopting the insider and outsider positions, the researcher was also able to objectively guide the whole process of planning for the research, data collection, analysis, and reporting. Adopting different research positions as was in this study is reported to have a likelihood of discouraging participants from disclosing information [37]. This was however not vividly experienced in this study.

### Data analysis

Responses from the HN/HND graduates were downloaded in the form of a Microsoft Excel spreadsheet, reviewed, cleaned, and further explored inductively to identify and code emerging themes using the conventional content analysis technique. Further coding was done to create additional sub-themes from the main themes. The process of coding and the subsequent creation of themes and subthemes were stopped when no more of these could be generated from the data. The created themes and sub-themes were then quantitized [38] into dichotomous variables of 0 or 1 depending on whether the coded response was absent or present in the individual participants’ narratives [39]. Upon quantitizing, the data set was exported to the IBM Statistical Package for Social Scientists software version 20 for analysis. Descriptive statistics and multiple response analyses were conducted and results presented mainly in the form of percentage distributions. Despite the likely drawbacks in quantitizing qualitative data, this approach allowed for enumerating the percentage of respondents mentioning particular themes and subthemes [41] from the varied narrative responses.

For the qualitative data from the academic staff and HN/HND work/internship supervisors, interview responses were initially transcribed and cleaned in Microsoft Excel 2010. Upon cleaning, the datasets were exported to the NVivo 12 Plus software where further exploration, coding, and content analysis were done using the same themes as those identified from the HN/HND responses. The coding of the different data sets was done by the principal researcher with support from two study assistants.

## Results

### Demographic, training, and occupational characteristics of the study respondents

#### HN/HND graduates

The demographics, training, and occupation characteristics of HN/HND graduates who responded to the survey are summarised in Table 1. Responses were received from 132 of the 450 invited participants; giving a general response rate of 29.3%. This response rate was higher than that usually obtained for online/email-based surveys [42]. Of the respondents, the majority were females (64.4%) and most (56.8%) were in the age category of 25–29 years.

**Table 1:** Demographic, Training and Occupation Characteristics of HN/HND Graduates

Characteristic	N	Category	***n***(%)
Gender	132	Female	85 (64.4)
		Male	47 (35.6)
Age categories	132	20–24	12 (9.1)
		25–29	75 (56.8)
		30–34	31 (23.5)
		35–39	11 (8.3)
		40 and above	3 (2.3)
Institution attended	132	Kyambogo University	112 (84.8)
		Makerere University	20 (15.2)
Year of study completion	132	2013–2016	81 (61.4)
		2009–2012	36 (27.3)
		2005–2008	15 (11.4)
Post-graduate training	132	None	73 (55.3)
		Master’s Degree	31 (23.5)
		Post-Graduate Diploma/Certificate	26 (19.7)
		Doctorate	2 (1.5)
Category of the place of the first internship	127	Regional referral hospital	54 (42.5)
		National referral hospital	26 (20.5)
		District hospital	16 (12.6)
		Non-Government non-profit hospital	11 (8.7)
		Other Government hospital	4 (3.1)
		Private for-profit hospital	4 (3.1)
		Health Center IV*	3 (2.4)
		Health Center III**	3 (2.4)
		Non-Government organization	3 (2.4)
		Research-based institution	2 (1.6)
		Donor project	1 (0.8)
Region where the first internship was undertaken	127	Central	70 (55.1)
		Western	24 (18.9)
		Eastern	18 (14.2)
		Northern	15 (11.8)
Category of the second place of internship	106	Non-Government organization	32 (30.2)
		Regional referral hospital	17 (16.0)
		District hospital	11 (10.4)
		Donor project	10 (9.4)
		Non-Government non-profit hospital	7 (6.6)
		Health Center IV*	6 (5.7)
		Government Ministry Department/Agency	6 (5.7)
		Private for-profit hospital	6 (5.7)
		National referral hospital	5 (4.7)
		Research-based institution	5 (4.7)
		Other Government hospital	1 (0.9)
Region where the second internship was undertaken	105	Central	63 (60.0)
		Western	21 (20.0)
		Northern	12 (11.4)
		Eastern	9 (8.6)
Category of current place of employment	115	NGO	70 (60.9)
		Health Facility	14 (12.2)
		District Local Government	8 (7.0)
		Academia	7 (6.1)
		Donor Agency/United Nations	6 (5.2)
		Government MDAs	6 (5.2)
		Industry	4 (3.5)
Region of current employment	115	Northern	40 (34.8)
		Central	39 (33.9)
		Western	18 (15.7)
		Outside Uganda	11 (9.6)
		Eastern	7 (6.1)

*HN/HND* Human Nutrition/Human Nutrition and Dietetics, *MDAs* Ministries, Departments, and Agencies.

* Health Centre IV: A level IV primary care facility in Uganda is one immediately below a district hospital; targets 100,000 people; acts as a referral facility for lower primary care facilities under its jurisdiction; has provisions for in-patient and laboratory services, and an operating theatre. It is usually staffed by qualified clinical officers, nurses, nurse aides, and doctors [40]. ** Health Centre III: In Uganda, a Health Centre III is a mid-level primary care facility; has provisions for basic laboratory services, maternity care, and inpatient care (often for onward referral); and is usually staffed by nurse aides, qualified nurses, and clinical officers [40]

In regards to training, the majority of respondents (84.8%) obtained their HN/HND Bachelor’s degree from Kyambogo University and most (61.4%) completed their studies in the years 2013–2016. Close to half of the respondents (44.7%) had undertaken further training at different levels; post-graduate diploma/certificate (19.7%), master’s degree (23.5%), and Ph.D. (1.5%).

In regards to places of internship, regional referral hospitals and the national referral hospital were the main places attended during the first internship at 42.5 and 20.5% respectively. On the contrary, non-government organizations and regional referral hospitals were mainly attended for the second internships at 30.2 and 16% respectively. As per the regions of Uganda where internships were undertaken, most respondents undertook their first internship in the central region (55.1%) followed by the western region (18.9%). The same trend was observed for the second internship with 60 and 20% of respondents respectively.

As for current employment; the majority of the respondents (60.9%) were employed by non-governmental organizations and the minority by healthcare-related facilities (12.2%). The northern and central regions of Uganda had the most graduates employed at 34.8 and 33.9% respectively. A small percentage of graduates (9.1%) reportedly worked outside Uganda.

#### Academic staff and work/internship supervisors

A total of 14 academic staff and 11 work/internship supervisors were interviewed in this study as shown in Table 2. The majority (64.3%) were females, 57.1% worked in Kyambogo University, 57.1% held doctorate level of training, and 42.9% had more than ten years of lecturing experience. On the other hand, 63.6% of the interviewed work/internship supervisors were males, 63.6% worked in regional referral hospitals, and 54.5% were in the position of a senior nutritionist.

**Table 2:** Demographic Characteristics of Interviewed Academic Staff and Work/Internship Supervisors

Participant Category	N	Characteristic	Category	*n*(%)
Academic staff	14	Gender	Female	9 (64.3)
			Male	5 (35.7)
		University	Kyambogo	8 (57.1)
			Makerere	6 (42.9)
		Level of Training	Doctorate	8 (57.1)
			Masters	6 (42.9)
		Years of Lecturing Experience	≥10 years	6 (42.9)
			6–10 years	4 (28.6)
			≤ 5 years	4 (28.6)
Work/internship supervisors	11	Gender	Male	7 (63.6)
			Female	4 (36.4)
		Category of Employer	Regional referral hospital	7 (63.6)
			Non-government organisation	4 (36.4)
		Work Position	Senior Nutritionist	6 (54.5)
			Program Manager	2 (18.2)
			Nutritionist	2 (18.2)
			Director	1 (9.1)

*HN/HND* Human Nutrition/Human Nutrition and Dietetics.

### Stakeholders’ perceptions of the nutrition and dietetics challenges faced by communities

As summarised in Table 3, the top five challenges mentioned by the HN/HND graduates included malnutrition in its different forms (60.5%); poor nutrition knowledge (43%); food insecurity (37.7%); non-communicable/chronic diseases (25.4%); and undesirable cultural and religious beliefs and practices (14.9%). Other challenges mentioned by the graduates included low dietary diversity (10.5% respondents); misleading information on nutrition (9.6%); inadequate maternal, infant, young child, and adolescent nutrition feeding practices (7.9%); and inadequate water, sanitation, and hygiene practices (7%).

**Table 3:** Stakeholders’ Perceptions on the Nutrition and Dietetics Related Challenges Faced by Communities

Graduates Perceptions (***N*** = 114)		Academic Staff and Work/internship Supervisors Perceptions
Nutrition and dietetics related challenges	Respondents ***n***(%)*	Illustrative quotes
Malnutrition in its different forms	69(60.5)	“There is a high prevalence of acute and chronic malnutrition in addition to other diseases …” “Some clients present with obesity, diabetes, asthma, cancer, and constipation. We see people of three categories; those that come for prevention, those who seek curative services, and those seeking treatment for infectious conditions”. “Disability due to micronutrient deficiencies, severe underweight, high stunting, wasting, anaemia, …”
Poor nutrition knowledge	49(43.0)	“Some community members lack knowledge on which foods to eat, how to prepare food, and on the frequency of feeding …” “Lack of extensive knowledge on cause-effect relation of disease and malnutrition …”
Food insecurity	43(37.7)	“90% of all the problems are because of food [insecurity] …” “Limitations in food access and availability …” “… household food insecurity …”
Non-communicable/chronic diseases	29(25.4)	“Non-communicable disease rates …”
Undesirable cultural and religious beliefs and practices	17(14.9)	“Cultural beliefs and food taboos …”
Low dietary diversity	12(10.5)	“Very few families can afford to eat the recommended minimum dietary diversity …” “Most people only have two meals a day and children are fed the same number of times as adults …”
Misleading information on nutrition	11(9.6)	“Limited access to information on nutrition & dietetics …”
Inadequate maternal, infant, young child, and adolescent nutrition feeding practices	9(7.9)	“… most children are weaned early and left under the care of their grandparents …” “… poor maternal and child health care …”
Inadequate water, sanitation, and hygiene practices	8(7.0)	“Poor hygiene and sanitation …”
Insecurity and economic related challenges	7(6.1)	“… sociocultural economic challenges …” “… border insecurity and cattle rustling by the Turkana …”
Limited access to and utilization of land for production	6(5.3)	
Climatic changes	5(4.4)	“… drought [and] occasional floods …”
Poor post-harvest handling and food quality control practices	2(1.8)	

*Multiple response analysis with percentage computed based on number of respondents rather than the total number of responses; *HN/HND* Human Nutrition/Human Nutrition and Dietetics

Based on the illustrative quotes by the academic staff and the work/internship supervisors as further summarised in Table 3, the nutrition-specific challenges were related to the high prevalence of malnutrition in its different forms; low dietary diversity as “very few families can afford to eat the recommended minimum dietary diversity …” ; and inadequate maternal, infant, young child, and adolescent feeding due to the observation that “… most children are weaned early and left under the care of their grandparents …” Some of the nutrition-sensitive challenges were observed to be related to food insecurity due to “limitations in food access and availability …” ; poor nutrition knowledge given that “some community members lack knowledge on which foods to eat, how to prepare food, and on the frequency of feeding …” ; undesirable cultural and religious beliefs and practices; and misleading nutrition information due to “limited access to information on nutrition & dietetics …”.

### Stakeholders’ perceptions of the nutrition and dietetics services requested for and provided to communities

Graduates’ responses were grouped under seven major domains as summarised in Table 4. Under the category of services requested by communities, responses were received from 109 graduates with the main services falling in the domains of nutrition awareness, education, and counselling (55%); integrated management of acute malnutrition (46.8%); food security and livelihoods support (33.9%); management of non-communicable diseases (26.6%); nutrition screening (22.9%); maternal, infant, young child, and adolescent nutrition (17.4%); and water, sanitation and hygiene (10.1%).

**Table 4:** Stakeholders’ Perceptions of the Demanded and Provided Nutrition and Dietetic Services

Nutrition and dietetic services domains	HN/HND Graduates			Academic staff and Work/internship Supervisors
	Requested by communities (***N*** = 109)	Provided by employers (***N*** = 100)	Considered a priority to provide (***N*** = 98)	
	Respondents *n*(%)*	Respondents *n*(%)*	Respondents *n*(%)*	Illustrative quotes on the nutrition and dietetic services provided in communities
Nutrition Awareness, Education & Counselling	60(55)	56(56)	63(64.3)	“Guidance and counselling …” “Nutrition guidance and counselling …” “Mainly nutrition education is provided …” “Nutrition education in the community …”
Integrated management of acute malnutrition	51(46.8)	54(54)	31(31.6)	“It is mainly the treatment of severe acute malnutrition through hospitals …” “Supplementation with ready-to-use-therapeutic feeds for those that meet the required criteria …” “Therapeutic feeding …”
Food Security and livelihood support	37(33.9)	47(47)	41(41.8)	“Nutrition livelihood programs …” “Food relief …” “Cash for work intervention …”
Management of non-communicable diseases	29(26.6)	22(22)	12(12.2)	“Interventions that largely address management of nutrition-related problems …” “Nutrition in illness in fairly larger hospitals and regional referral hospitals …” “Diet/meal planning …”
Nutrition Screening	25(22.9)	28(28)	7(7.1)	“Nutrition assessment …” “Routine community nutrition screening for children …”
Maternal, infant, young child and adolescent nutrition	19(17.4)	19(19)	14(14.3)	“Child health nutrition services at clinics and hospitals …” “Antenatal nutrition education …” “Food demonstration in infant and young child feeding …” “Health education on infant and young child feeding practices …”
Water, sanitation, and hygiene	11(10.1)	14(14)	8(8.2)	“Water sanitation and hygiene services”
Business and industry	_	_	_	“Product development, food processing …”

* Multiple response analysis with percentage computed based on number of respondents rather than the total number of responses; *HN/HND* Human Nutrition/Human Nutrition and Dietetics

For the nutrition and dietetic services provided by employers, the majority of respondents (56%) mentioned nutrition awareness, education, and counselling services as being the main provided services; followed by integrated management of acute malnutrition (54%); food security and livelihood support (47%); nutrition screening (28%); management of non-communicable and communicable diseases (22.0%); maternal, infant, young child and adolescent nutrition (19%); and water, sanitation and hygiene (14%).

When asked about their views on the would-be priority nutrition and dietetics services to provide, most graduates (64.3%) mentioned services under the domain of nutrition awareness, education, and counselling; followed by food security and livelihood support (41.8%); integrated management of acute malnutrition (31.6%); maternal, infant, young child, and adolescent nutrition (14.3%); management of non-communicable and communicable diseases (12.2%); and water, sanitation and hygiene (8.2%). Some illustrative quotes to justify the responses are summarised along with the responses in Table 4.

The responses by the academic staff and the work/internship supervisors in regards to the nutrition and dietetic services provided in communities were coded under similar themes as those used for the HN/HND graduate as also summarised in Table 4. Based on the illustrative quotes, under the theme of management of non-communicable and communicable diseases; respondents noted the provision of interventions that largely address the management of nutrition-related problems by ‘fairly’ large hospitals and referral hospitals. Relatedly, services in regards to the treatment of severe acute malnutrition, therapeutic feeding, and supplementation with Ready-to-use-therapeutic feeds for those that meet the required criteria were perceived to be provided under the theme of integrated management of acute malnutrition.

Under the theme of food security and livelihood support; mention was made for the provision of nutrition livelihood programs, food relief, and cash for work interventions. Some of the services perceived to be provided under the theme of maternal, infant, young child, and adolescent nutrition included child health and nutrition services in clinics, antenatal nutrition education, food demonstrations in infant and young child feeding, and health education. Under the theme of nutrition screening, mention was made of nutrition assessment and routine community nutrition screening for children. Some of the services mentioned under the theme of nutrition awareness, education, and counselling included guidance and counselling and community nutrition education. Other mentioned services included water, sanitation, and hygiene; and product development and food processing under the themes of water, sanitation, and hygiene, and business and industry respectively.

### Stakeholders’ perceptions of the nature of work and job roles performed by practicing HN/HND graduates

As per the graduates’ responses, the nature of work done by the practicing graduates was categorised under seven major domains (see Table 5) of organizational leadership and management (69.6%); management of nutrition-related disease conditions at health facility and or community level (59.1%); nutrition and health promotion (57.4%); research and documentation (53%); advocacy, communication and awareness (23%); academia (7.1%); and that of the business/industry (3.6%).

**Table 5:** Categories of Work and Related Roles Performed by HN/HND Professionals Practicing in Uganda

Categories of Work			Identified Work/Job Roles		
**Category of Work Domain**	***N***	**Graduates whose work relates to domain** ***n*(%)**	**Work roles**	***N***	**Graduates performing mentioned role** *n*(%)*
Organizational Leadership and Management	115	80(69.6)	Leadership	80	60(75)
			Project planning, management, & implementation		43(53.8)
			Project monitoring and evaluation	79	24(30.4)
			Budgeting and accountability	80	18(22.5)
			Human resources management		15(18.8)
			Organizational representation and networking		13(16.3)
			Proposal/report writing	79	5(6.3)
			Resource mobilization	80	3(3.8)
			Technical support/guidance	79	3(3.8)
Management of Nutrition Conditions at Health Facility and or Community level	115	68(59.1)	Nutrition screening or assessment	67	51(76.1)
			Inpatient therapeutic care		39(58.2)
			Outpatient therapeutic care		39(58.2)
			Supplementary feeding		35(52.2)
			Nutrition education and counselling	66	26(39.4)
			Nutrition supplies management/procurement	67	14(20.9)
			Monitoring, supervision, and QI		13(19.4)
			Community nutrition work		11(16.4)
			Management of other nutrition-related disease conditions	66	5(7.6)
			Emergency nutrition	66	4(6.1)
			MIYCAN	67	4(6.0)
Nutrition and health promotion	115	66(57.4)	Nutrition and health education	66	41(62.1)
			Capacity building	65	34(52.3)
			Technical support		12(18.5)
			Growth monitoring and promotion	66	2(3)
Research and documentation	115	61(53)	Dissemination	61	61(100)
			Report writing		45(73.8)
			Undertaking research		28(45.9)
			Data entry and analysis		5(8.2)
			Proposal writing		4(6.6)
Advocacy, Communication, and Awareness	113	26(23)	Community/stakeholder engagements	26	20(76.9)
			Stakeholder orientation		2(7.7)
			Networking		2(7.7)
			Resource mobilization		1(3.8)
			Coordinating advocacy events		1(3.8)
Academia	112	8(7.1)	Lecturing and student supervision	8	87.5
			Research	8	12.5
Business/Industry	112	4(3.6)	Product development	4	1(25)
			Product marketing	4	1(25)
			Technical Assistance	4	1(25)
			Product certification	4	1(25)

* Multiple response analysis with percentage computed based on number of respondents rather than the total number of responses; *HN/HND* Human Nutrition/Human Nutrition and Dietetics**,** *MIYCAN* Maternal, infant, young child, and adolescent nutrition, *QI* quality improvement

For the respondents whose work entailed aspects of organizational leadership and management; the main mentioned roles included leadership (75%); project planning, management and implementation (53.8%); project monitoring and evaluation (30.4%); budgeting and accountability (22.5%); and human resources management (18.8%).

For the respondents with job roles categorized under the domain of management of nutrition conditions at the health facility and community level, the main mentioned work roles included nutrition screening or assessment (76.1% of respondents); offering of inpatient and outpatient therapeutic care each at 58.2%; supplementary feeding (52.2%); nutrition education and counselling (39.4%), nutrition supplies management (20.9%); monitoring, supervision and quality improvement (19.4%); and community nutrition work (16.4%).

The main work roles performed by graduates engaged in nutrition and health promotion included nutrition and health promotion (62.1%); capacity building (52.3%); offering technical support (18.5%); and growth monitoring and promotion (3%).

For undergraduates engaged in research and documentation, all (100%) reportedly participated in dissemination; followed by 73.8% that undertook report writing, 45.9% that participating in carrying out nutrition surveys, 8.2% that did data entry and analysis, and 6.6% that contributed to proposal writing.

For the undergraduates whose job roles were categorized as falling in the domain of advocacy, communications, and awareness, the majority (76.9%) mentioned carrying out community/stakeholder engagements. This was followed by undertaking stakeholder orientations and networking each at 7.7% and coordinating advocacy events (3.8%).

Besides the feedback from the HN/HND graduates, this study also explored the views of academic staff and those of the work/internship supervisors on the types of employment in which the HN/HND graduates were engaged as well as the work/job roles they performed. From the responses, it was evident that employed graduates mainly worked as nutritionists in different positions; across different sectors; and in both government and non-government agencies. As per the following respondents’ narratives, the ministry of health, non-governmental organizations, academic institutions, private health facilities, United Nations agencies, hotels, district local governments, and special care homes included some of the mentioned employers.

“They are employed in the public service as nutritionists at the national level, in the ministry of health, in regional referral hospitals, and at local government level …. [They] also work as nutrition specialists with various NGO’s and United Nations bodies.”

“Nutritionists in humanitarian relief based organizations; nutrition program managers in NGOs; nutritionists and dieticians in the national referral hospital, district hospitals, and private hospitals; nutrition and diet consultants; lecturers; and researchers.”

The graduates were regarded to be performing multiple cross-cutting roles in the different types of employment. For instance, one of the respondents noted the HN/HND job roles to involve “Conducting nutrition education for various groups; individual nutrition counselling sessions for various nutrition-related conditions; dietary assessment; conducting of food demonstration sessions in infant and young child feeding; and nutrition project planning and implementation.”

The performed roles were however observed by one of the respondents to vary according to the job position, “Roles vary depending on the rank. Supervisory work is done as one grows in rank; other roles assigned depending on rank include nutrition assessment; … undertaking nutrition interventions for clients; giving food supplements, and client follow up.”

### Stakeholders’ perceptions of the knowledge and skills required of HN/HND graduates in Uganda

From the multiple responses by the HN/HND graduates, 20 knowledge and skills themes were identified. Based on these themes; an evaluation of the respondents’ perceptions in regards to knowledge and skills expected of HN/HND graduates; knowledge and skills gaps amongst individual graduates; knowledge and skills inadequately attained while at university; and the knowledge and skills recommended as a training minimum for HN/HND was done as summarised in Table 6.

**Table 6:** HN/HND Graduates' Perceptions of the Knowledge and Skills Required for Health Systems Performance

Knowledge & Skills Themes	Expected of HN/HND Graduates (***N*** = 132)	Gaps Amongst Individual Graduates (***N*** = 120)	Inadequately Attained while at University (***N*** = 100)	Recommended as Training Minimum (***N*** = 97)
	**Respondents** *n*(%)*	**Respondents** *n*(%)*	**Respondents** *n*(%)*	**Respondents** *n*(%)*
Integrated management of acute malnutrition	100(75.8)	38(31.7)	13(13)	40(41.2)
Communication, education, and counselling	82(62.1)	22(18.3)	13(13)	27(27.8)
Medical/clinical nutrition therapy	75(56.8)	74(61.7)	61(61)	43(44.3)
Research, data analysis, proposal, and report writing	69(52.3)	12(10.0)	23(23)	19(19.6)
Leadership and management	62(47)	14(11.7)	19(19)	19(19.6)
Nutrition screening and assessment	63(47.7)	13(10.8)	8(8)	25(25.8)
Project planning, management, monitoring, and evaluation	49(37.1)	46(38.3)	17(17)	6(6.2)
Nutrition Advocacy	46(34.8)	12(10)	10(10)	13(13.4)
Maternal, infant, young child, and adolescent nutrition	43(32.6)	7(5.8)	1(1)	9(9.3)
Nutrition computing	32(24.2)	8(6.7)	11(11)	11(11.3)
Agribusiness	11(8.3)	2(1.7)	–	3(3.1)
Public health	9(6.8)	1(0.8)	1(1)	3(3.1)
Quality improvement	9(6.8)	–	4(4)	–
Product development and food safety management	9(6.8)	6(5.0)	4(4)	–
Records and data management	6(4.5)	–	–	–
Emergency nutrition	2(1.5)	1(0.8)	1(1)	–
Water, sanitation, and hygiene	2(1.5)	–	–	2(2.1)
Professional ethics	1(0.8)	4(3.3)	–	–
Internal medicine	–	1(0.8)	–	–
Human anatomy	–	–	7(7)	–

* Multiple response analysis with percentage computed based on number of respondents rather than the total number of responses; *HN/HND* Human Nutrition/Human Nutrition and Dietetics

In regards to the knowledge and skills expected of HN/HND graduates, responses were received from 132 respondents. The top six mentioned themes were integrated management of acute malnutrition (75.8%); communication, education, and counselling (62.1%); medical/clinical nutrition therapy (56.8%); research, proposal, and report writing (52.3%); nutrition screening and assessment (47.7%); and leadership and management (47%).

For the knowledge and skills gaps amongst individual graduates; responses were received from 120 graduates. The top six mentioned gaps included medical/clinical nutrition therapy (61.7%); project planning, management, monitoring and evaluation (38.3%); integrated management of acute malnutrition (31.7%); communication, education, and counselling (18.3%); leadership and management (11.7%); and research, data analysis, proposal and report writing (10%).

Concerning the knowledge and skills not adequately attained during undergraduate training, feedback was received from 100 respondents. The top six knowledge and skills themes were medical/clinical nutrition therapy (61%); research, data analysis, proposal and report writing (23%); leadership and management (19%); project planning, management, monitoring and evaluation (17.6%); integrated management of acute malnutrition; and communication, education, and counselling (13% each); and nutrition advocacy (10%).

As relates to the knowledge and skills recommended for minimum training, 97 respondents provided feedback. The top recommended knowledge and skills themes were in the categories of medical/clinical nutrition therapy (44.3%); integrated management of acute malnutrition (41.2%); communication, education, and counselling (27.8%); nutrition screening and assessment (25.8%); leadership and management (19.6%), and nutrition advocacy (13.4%).

Similar to the responses by HN/HND respondents, the perceptions of academic staff and work/internship supervisors were multifaceted. Illustrative quotes of the academic staff and work/internship supervisors’ responses when coded to the same knowledge and skills domains as was for the HN/HND respondents are summarised in Table 7. Under agribusiness, the need for knowledge and skills in crop management and backyard farming was mentioned. Under medical/clinical nutrition therapy, it was expressed that Nutrition and Dietetics professionals needed knowledge and skills on how to assess, categorize and apply diet therapy to correct and manage disease abnormalities and dietary recommendations for specific age groups. Relatedly, HN/HND graduates were noted to have knowledge and skills gaps in clinical care and support for clients; management of clients in the absence of medical officers; management of non-communicable diseases; making of proper diagnosis; and dietetic management.

**Table 7:** Academic staff and Work/internship Supervisors' Perceptions on the Knowledge and Skills Required of HN/HND Graduates

Knowledge and Skills Domain	Illustrative quotes	
	Required of Nutrition and Dietetics Professionals in Uganda	Knowledge and Skills Gaps
Agribusiness	“Crop management, backyard farming …”	
Medical/clinical nutrition therapy	“How to apply diet therapy to correct or manage abnormalities …” “Dietetic management of some disease conditions …” “Prevention of overweight and obesity …” “Special preparation food skills for various age groups and health conditions …” “Assessment, categorization, and management of patients using therapeutic feeds …” “Dietary recommendations for specific age groups …”	“Clinical care and support for clients …” “Most lack the ability to manage a client in the absence of a medical officer …” “Nutrition management of non-communicable diseases …” “Inability to make a proper diagnosis of a disease … and manage it dietetically …”
Nutrition computing	“Use of technology …” “Basic computing …” “Data analysis …”	“Inadequate exposure to the use of modern technology …” “Statistics was not well mastered …”
Maternal, infant, young child, and adolescent nutrition	“Integrated management of acute malnutrition; infant and young child feeding … nutrition assessment, counselling, and support …” “Infant and young child feeding …”	“Infant and young child feeding …”
Leadership & Management	“Team working skills, good interpersonal, … organizational skills” “Critical thinking …” “Ability to execute … duties with minimum or no supervision”	“Lack of leadership and governance skills …” “Conflict management, negotiation skills, strategies to boost and sustain the performance of the organization and its employees …”
Communication, education, and counselling	“Nutrition education and counselling …” “Facilitation skills since they are working with people …” “Communication skills … but also need skills that are more specific to behavioural change” “Nutrition education and advocacy …”	“Communication skills, interpersonal skills …” “Confidence talking to the public …” “Education session planning and implementation” “Inability to effectively communicate with clients …” “Development of IEC materials …” “Presentation skills …”
Nutrition screening and assessment	“Nutrition assessment …” “Nutrition assessment i.e. anthropometry, biochemical, clinical, and dietary assessment for all people …”	“Nutrition assessment …”
Project planning, management, monitoring, and evaluation	“Planning, budgeting, policy formulation and dissemination, nutrition governance …” “Understanding of multi-sectoral nutrition programming …” “Project implementation …”	“Monitoring and evaluation …” “Project planning …” “Budgeting …” “Writing bidding proposals for organizations …”
Research, Proposal, & Report writing	“Research both field-based and laboratory-based …” “Data analysis and report writing …” “Analytical skills to be able to carry out research and analyse different problems …” “Data collection, statistical analysis, and interpretation …”	‘Proposal writing …” “General research knowledge …” “Report writing and data collection …”
Product development and food safety management	‘Quality control analysis …” “Food safety in nutrition …” “Food standards and laws …”	
Professional Ethics	“Ethics and professionalism …”	“Work ethics, Client management …” “Ethics and professionalism …”
Anatomy, Physiology, Pharmacology, Pathology, Biochemistry	“Anatomy …” “Drug prescription with minimal reliance on medical doctors …” “Understanding of the relationship between foods and the blood system …” “Pharmacy and pathology …” “Biochemistry and food microbiology …”	“Linking nutrition and body system functions …” “Anatomy and physiology …”
Laws, policies and regulations	“Knowledge and skills of key policies and guidelines that guide nutrition …”	“Nutrition policy and legislation …”

*HN/HND* Human Nutrition/Human Nutrition and Dietetics.

Under the domain of nutrition computing, it was expressed that undergraduates needed to have the knowledge and skills necessary for the use of technology, basic computing, and data analysis. Knowledge and skills gaps were noted to exist in the form of inadequate mastery of statistics and limited exposure to the use of modern technology.

Under the broad domain of maternal, infant, young child, and adolescent nutrition, stakeholders expressed a need for knowledge and skills in infant and young child feeding, as well as knowledge and skills in specific areas of maternal, infant, young child, and adolescent nutrition. However, some respondents noted the existence of knowledge and skills gaps in infant and young child feeding.

In the domain of leadership and management, knowledge and skills were noted to be required in team working skills, interpersonal and organizational skills; critical thinking; and the management of human and material resources. Respondents noted the existence of knowledge and skills gaps in leadership and governance, conflict management and negotiation, and mobilization and fundraising.

Some of the knowledge and skills noted as being required in the domain of communication, education, and counselling included nutrition education and counselling, facilitation skills, communication more so as relates to behavioural change, and nutrition education and advocacy. Noted knowledge and skills gaps existed in communication skills and interpersonal skills, confidence talking to the public, education session planning and implementation, inability to effectively communicate with clients, development of IEC materials, and presentation skills.

Under the domain of nutrition screening and assessment, knowledge and skills were said to be expected of undergraduates in nutrition anthropometry, biochemical, clinical, and dietary assessment. The undergraduates were however said to have inadequate skills in nutrition assessment.

In the domain of project planning and management, knowledge and skills were said to be expected of undergraduates in planning, budgeting, policy formulation, and nutrition governance; understanding of multisectoral nutrition programming, and project implementation. Notable knowledge and skills gaps existed in monitoring and evaluation, project planning, budgeting, and writing bidding proposals for organizations.

The expected knowledge and skills under the domain of research, data analysis, proposal, and report writing related to undertaking field and laboratory-based research; statistical data analysis and interpretation; report writing; and analytical skills. However, knowledge and skills gaps were said to exist in different areas including proposal writing, general research knowledge, and report writing and data collection.

In the domain of anatomy, physiology, pharmacology, pathology, and biochemistry, respondent expressed a need for knowledge and skills in anatomy, drug prescription with minimal reliance on medical doctors, understanding of the relationship between foods and the blood system, pharmacy and pathology, and biochemistry and food microbiology. Some noted knowledge and skills gaps were in the ability to link nutrition and body system functions, and limitations in anatomy and physiology.

In the broad domain of product development and food safety management, knowledge and skills were expected of undergraduates in quality control analysis, food safety in nutrition, and food standards and laws.

Under the domain of professional ethics, respondents indicated the need for undergraduates to exhibit knowledge and skills in ethics and professionalism. However, knowledge and skills gaps were noted in work ethics and client management, and general ethics and professionalism.

An extra domain of laws, policies, and regulations was created based on the trainer’s and supervisors’ responses. Under this domain, Nutrition and Dietetics professionals were expected to exhibit an understanding of key policies and guidelines on nutrition at the global and national levels. However, it was echoed that HN/HND professionals exhibited knowledge and skills gaps in nutrition policy and legislation.

### Observations from the stakeholder validation workshop

A one-day results validation workshop was undertaken on the 3rd of October 2019 at the Grand Global Hotel located in Makerere Kikoni, Kampala Uganda. The workshop aimed to engage stakeholders in reviewing and commenting on the appropriateness of the study results. The workshop followed a predefined formal agenda that was communicated to the participants before the workshop. Key on the agenda was the oral presentation of the study background, methodology, and results by the principal researcher; plenary reactions, discussions, and suggestions in regards to the presented content; and three group exercises each followed by a plenary presentation and discussions. To document workshop proceedings, three experienced note-takers were engaged in taking notes; audio recording of the plenary presentations and discussions was done, and photographs were taken at different intervals. The validation workshop was attended by 31 participants (one from the food/business industry, five working with private hospitals, 12 working with universities, two working with district local governments, six working with NGOs, one working with a research-based institution, two working with regional referral hospitals, one working as a private consultant, and one working with the ministry of health).

In general, the participants judged the study to be vital and timely towards informing on-going processes in the development of a national HN/HND training and practice standard that was considered inexistent at the time of the study; “Uganda has no National standard stipulating the training and practice requirements for nutrition and dietetics …. We hope this research can be used to inform such process”. One of the participants sought clarification on how the study links and informs curriculum reforms; “At what point do the study findings link with the curriculum; is it the issue of competencies not being in the curriculum or is it due to how the teaching is done? How do we link these?”

Participants further recognised the multisectoral nature of implementing nutrition interventions at the global and national level and called for the need to take caution when identifying the knowledge and skills required of HN/HND graduates to perform in a multisectoral setting:

“Issues like water, sanitation, and hygiene; food security; and livelihood support as reported in the findings are very key as they introduce an element of multi-sectorality … caution needs to be undertaken in regards to the nutrition and dietetics training needs under the multisectoral approach. We must be cautious about the knowledge and skills required under the multisectoral setting.”

Relatedly, the need to appreciate the roles of other professions in the multisectoral approach; linking with them, and understanding their views in regards to the knowledge and skills they expect of HN/HND professionals was emphasised:

“We should appreciate that other professions providing services that are supportive of nutrition and dietetics services do exist. We need to know how to link with them to implement multisectoral nutrition and dietetics services.”

“We need to know how other professionals perceive of our profession, and the knowledge and competencies they expect of us …. Coming from a clinical and research setting, I sense others don’t know what to expect of nutritionists. Can we collect additional data on this? Getting the perceptions of other professionals that work with nutritionists and dietitians on what they expect of nutritionists and dietitians can enable us to make better curriculum reforms in nutrition and dietetics training.”

In discussing the knowledge and skills required for the practice of HN/HND in Uganda, observations were made that some knowledge and skills areas had been lowly ranked:

“Why was the response rate on professional ethics low? Is it because adherence to ethics is low in professional nutrition and dietetics practice or is it because nutrition and dietetics professionals do not value ethics? The curriculum needs to fully equip trainees with professional ethics because there is still a challenge on which ethics guide nutrition and dietetics practice in Uganda.”

“Patient clerking was missed out in the results yet it is an important competency that has to be mastered by nutritionists and dietitians …”.

Some participants also argued that graduates exhibited minimal competency in some of the areas considered core for the practice of HN/HND in Uganda.

“Nutrition in emergencies is not well taught in Uganda to Bachelor’s students of nutrition yet many undergraduates work in emergency contexts and or with humanitarian agencies whose work entails aspects of emergency nutrition …”.

“The current transmission of knowledge is insufficient … fresh undergraduates severely lack practical clinical skills …”.

“The undergraduates we have can generate statistics but not use the information yet professionals should be able to interact with the information, generate evidence to guide programming … statistics are basic at the undergraduate level but undergraduates need more than is stipulated for this level …. [Graduates] don’t appreciate the use of statistics to guide programming, this is reflected when they start working.”

“Knowledge and skills need to be tailored to the environment in which the undergraduates operate. What challenges are undergraduates facing in the environments they are operating? We should consider the policy and planning environment because these affect professional work.”

The need to introduce and or strengthen training in family planning, procurement, leadership, and nutrition policy formulation was recommended:

“It may be important to consider including family planning, strengthening training in procurement and logistics of nutrition materials and supplies, and adding nutrition leadership and management as these are lacking in the HN/HND Bachelors training curriculum.”

“Many of the challenges presented are in the services sector but not in policy yet [many] challenges exist in the implementation of nutrition-related policies. Nutrition and dietetics related challenges presented have limited focus on policies being implemented yet the government, in terms of systems capacity, under the Ministries of Health and Agriculture focuses on implementing what is stipulated in the policies. I suggest for the need to undertake further reflection on nutrition-related policies in the country and the capacity of concerned ministries to implement nutrition as stipulated in the existing policies and sectoral strategies …”.

“There is a need to improve utilization of HMIS [Health Information Management Systems] data collected from the community by nutritionists.”

The need to undertake assessments to determine the capacity readiness of institutions of higher learning to train HN/HND in Uganda was also emphasised; “Have you considered looking at the capacity of the different institutions training nutrition undergraduates in Uganda in terms of research, equipment, and human resources …? ”.

## Discussion

This study used a cross-sectional mixed methods design to assess the nutrition and dietetic needs and the requisite professional competencies in Uganda. Participants included graduates of the Bachelors in HN/HND respectively obtained from the Makerere and Kyambogo Universities of Uganda in the period 2005–2016; academic staff that train HN/HND in two universities; and work/internship supervisors of the HN/HND graduates. The majority of the HN/HND respondents graduated in the years 2013–2016. The academic staff and HN/HND work/internship supervisors that participated in the study had varied experiences; academic and non-academic. Besides the Universities, non-academic institutions including hospitals, government agencies, and non-governmental organisations were reported to have been instrumental in the training of nutrition and dietetics through offering internship opportunities to HN/HND study interns. Most graduates undertook study internships in non-government organizations, regional and national referral hospitals in the central region of Uganda. Less than half of the graduates had undertaken additional training; those who had done so had mainly obtained master’s level training. Graduates were employed by government and non-government organisations across the different regions of Uganda; with some employed outside the country. Non-governmental organisations were the main employers of HN/HND graduates with the northern and central regions of Uganda having the most concentration of employed graduates.

Stakeholders generally indicated that communities faced a variety of nutrition-specific and nutrition-sensitive challenges. Hence, the nutrition interventions requested, provided, and considered a priority to provide to communities were both nutrition-specific and nutrition-sensitive. Nutrition interventions in the areas of awareness, education, and counselling; integrated management of acute malnutrition; and food security and livelihoods were found to be the main ones demanded by communities, provided by employers, and also considered by the graduates as a priority to provide. The nature of work done by HN/HND graduates varied; with the dominant entailing aspects of organizational leadership and management; management of nutrition-related disease conditions both in health facilities and communities; nutrition and health promotion; research and documentation; and advocacy, communication, and awareness. About 20 knowledge and skills areas were identified as being required by HN/HND graduates to implement the different nutrition interventions in Uganda. Of these, integrated management of acute malnutrition; communication, education, and counselling; medical/clinical nutrition therapy; research, proposal and report writing; nutrition screening and assessment; and leadership and management comprised the top six mentioned as being expected of graduates. Although highlighted amongst the top knowledge and skills areas required of HN/HND graduates, medical/clinical nutrition therapy; integrated management of acute malnutrition; communication, education, and counselling; leadership and management; and research, data analysis, proposal and report writing also featured amongst the top knowledge and skills gaps amongst individual HN/HND graduates. HN/HND graduates further indicated not having adequately attained knowledge and skills in most of the key areas they are expected to be competent; pointing to a likely mismatch between the knowledge and skills expected of HN/HND undergraduates to those obtained through training.

Bruening et al. [43] noted that skills development in nutrition and dietetics requires professional training and supervised practice for one to progress from being competent to becoming an expert. The observations that some graduates had undertaken the initiative to undertake further formal training and that the regional and national referral hospitals and non-government organizations took on high numbers of student interns highlights three major aspects: (1) the need for professional growth and development by way of having tailored training programs through which HN/HND trainees can obtain experiential training to develop their professional competence profiles as well as obtain higher academic credentials; (2) the emerging relevancy of non-academic institutions to support HN/HND training in Uganda by offering experiential attachments to trainees; and (3) the value of interprofessional and inter-institutional collaborations in HN/HND training. The observation that most internships were undertaken in organizations located mainly in the central region and yet graduates worked across all the different regions of the country calls for initiatives to expose students to the cultural and social welfare conditions prevalent across all regions of the country during training. The variedness in the employment of HN/HND graduates to an extent shows a probable increase in employment opportunities for HN/HND professionals in sectors beyond health. This is probably due to the increased focus by different stakeholders, government, and non-government, to address nutrition multisectorally hence creating more demand for the HN/HND professionals in different sectors. The observation that most graduates were working in the northern region of the country could be due to the increased presence of NGOs operating in that region due to the influx of refugees. The northern region of Uganda had different refugee camps including the Bidi bidi refugee settlement which was considered one of the largest refugee settlements in the world [44]. Given that most organizations have their headquarters in the central region of Uganda; it was not surprising that the central region had the second-highest number of graduates employed.

Although the findings of this study are mainly based on the provider perspectives, this study reaffirms findings in other literature that communities across Uganda face multiple nutrition and dietetic challenges [14, 45, 46] that require to be addressed by different stakeholders, at different levels, through the implementation of both nutrition-specific and nutrition-sensitive interventions [14, 47, 48]. The stakeholders’ perceptions also justify earlier observation that communities face multiple nutrition and dietetics challenges that require a multiplicity of interventions to be addressed [49]. Worth noting also is that Uganda embraced the multisectoral model of implementing nutrition interventions through which different actors and sectors are mobilized to address malnutrition at different levels [14, 48, 50]. Given that awareness, education, and counselling are key in influencing behavioural change, findings of this study also substantiate earlier findings by Lubogo and Orach [51] that pointed to community and behavioural nutrition research as one of the most desirable options for addressing undernutrition in Uganda. The nature of work and the related job roles performed by employed HN/HND graduates to an extent mirrored those of similar professionals in other countries including the developed countries. For instance, Registered Dietitians in the United States work in various sectors including health care settings, community and public health care settings, academia, research, food, and nutrition industry, and the business industry [22]. Some work roles were so specific to nutrition and dietetics and hence required specialized training. Other roles, though not requiring specific training in nutrition and dietetics were core for the Nutrition and Dietetics professionals to master for the graduates to exhibit all-round competency in the profession. This observation points to the need to equip Nutrition and Dietetics professionals in Uganda with both profession-specific and interprofessional knowledge and skills. According to Frenk, Julio, Chen, et al., [20] profession-specific competencies can be attained through CBE. On the other hand, interprofessional competencies can best be attained through interprofessional education that involves students of two or more professions learning together about each other’s roles [20]. Adoption of interprofessional education in nutrition and dietetics training is important given that Nutritionists and Dietitians work in teams with other professionals.

Further review of the identified knowledge and skills themes reveals some similarities in the identified knowledge and skills themes to what has been proposed for degree-level training in the regional model nutrition curriculum for health workers based in the East, Central, and Southern Africa Health Community [52]. On the other hand, these findings could also be highlighting the existence of probable inefficiencies in the curricula and inadequacies in the capacity of higher education institutions to train competent HN/HND in Uganda. Based on these observations and bearing in mind that “Inadequate knowledge, skills, and inappropriate attitudes can all form obstacles to good health care” [53], we foresee a need for undertaking curricula and institutional reforms in the training of HN/HND in Uganda to ensure that graduates get equipped with the knowledge and skills required for the practice of nutrition and dietetics at the national, regional, and global level. It should be highlighted that the responses were obtained from HN/HND graduates that were trained using earlier versions of the curriculum. Kyambogo University, in particular, adopted a new four-year curriculum in 2016 to replace the three-year curriculum that it had been using. The highlighted inefficiencies could have been corrected in the revised curriculum. Hence, the HN/HND responses on the knowledge and skills not adequately obtained during training would likely differ if undergraduates trained using the recent curriculum for Kyambogo were interviewed. However, the fact that responses were obtained from practicing graduates gives a clear indication of the nutrition and dietetics related challenges addressed by the graduates; the knowledge and skills gaps amongst the graduates; and the knowledge and skills required of Nutrition and Dietetics professionals to practice in the varied health systems settings in Uganda.

The need for competent Nutrition and Dietetics professionals as highlighted in this study aligns with the aspirations by the Government of Uganda to train and equip graduates with the necessary skills and abilities to meet Uganda’s development needs [15, 16]. It is also in line with the African Union strategy to “Reorient Africa’s education and training systems to meet the knowledge, competencies, skills, innovation and creativity required to … promote sustainable development at the national, sub-regional and continental levels” [25]. In line with the recommendation to improve the training of health professionals for the twenty-first century [20], it is worth noting that countries are increasingly adopting CBE in the training of their Nutrition and Dietetics professionals to equip graduates with the knowledge and skills required for health systems performance [54–60]. The adoption of CBE in HN/HND training in Africa is however still low [19] but has since been embraced by countries like South Africa [61]. Research undertaken in Kenya showed that undertaking curricula review is critical to improving the quality and relevance of health workforce training [62]. Although the possession of knowledge and skills required for health systems performance is key for all health professionals [20], whether undergraduates can possess all the expected/required competencies by the time they graduate is contested. In the United States of America, the development of skills by Nutrition and Dietetics professionals was envisaged to occur in different phases; from one being a novice, progressing to the level of an advanced beginner, to becoming competent, proficient and in the end an expert [19, 43]. Based on this view, it can be assumed that defining who a competent Nutrition and Dietetics graduate is depends on what is stipulated in the existent national or institutional nutrition and dietetics training and practice standards/curriculum. However, there were no existent national specific training and practice standards/curricula for the HN/HND profession in Uganda at the time of undertaking this research.  As also observed in our earlier study [19], the curriculum used to train HN/HND in Uganda were institutional specific and mainly specified learning outcomes instead of the competencies that students are expected to exhibit upon graduation.

### Study implications

#### Education and practice

From a training and practice perspective, the finding that HN/HND professionals were working in different sectors, within and outside the country re-emphasises the multisectorality of nutrition and dietetics and highlights a need for HN/HND graduates to be equipped with the knowledge and skills required for working with multiple actors, in a multisectoral setting, within and outside Uganda. As such, the need to reorient the current University HN/HND training programs to ensure that graduates obtain the knowledge and skills required for the job market while creating opportunities for graduates to further obtain and develop professional competencies in nutrition and dietetics is pertinent. We recommend using the study findings as a basis for (1) undertaking further stakeholder dialogues towards developing consensus on the key competencies that should be exhibited by all HN/HND graduates in Uganda and (2) subsequently developing a competency framework that delineates the sets of competencies that should be acquired at different levels of HN/HND training. Such a framework, developed basing on national evidence with the input of different stakeholders, has the added advantage of limiting the wholesale adoption of HN/HND training models based on other country contexts [20] and may further promote efficient investments in HN/HND professional education in Uganda. The identified competencies and the related competency framework should contain knowledge and skills specific to nutrition and dietetics including but not limited to areas such as nutrition assessment; medical/clinical nutrition therapy; integrated management of acute malnutrition; and infant and young child feeding as well as other general knowledge and skills in areas such as communication, education, and counselling; leadership and management; research; project planning and management; and advocacy, and social behavioural change for these have been shown by this study to be essential for the practice of nutrition and dietetics in Uganda’s varied health systems settings.

#### Policy

As identified in this study, the training of HN/HND professionals in Uganda requires the engagement of academic institutions for the provision of specialised formal training and non-academic institutions for experiential attachments. As such, there is a need for higher academic institutions to form formal partnerships with non-academic institutions. Forming training partnerships has the advantage of fostering experiential student learning, closer monitoring, and supervision of what students are exposed to during internship training. The formation of such partnerships may however necessitate the setting up of standards or criteria based upon which non-academic institutions are qualified and selected to host HN/HND student interns. However, as is currently, the engagement of non-academic institutions is less formal and no set national standards/criteria exist to guide the selection of which institutions should be engaged in HN/HND internship placements. We recommend the need for a policy that mandates Universities to (1) work with the relevant ministries of health, education, and agriculture, and other non-state actors that support nutrition and dietetic services delivery to develop national HN/HND training and practice standards as well as (2) formalise internship relations through signing Memorandums of Understanding with organisations meeting set practicum standards. Such measures will foster closer collaboration between academic institutions and the different organisation that employ HN/HND graduates in Uganda with the added benefit of improving nutrition and dietetics training and service delivery.

#### Research

Further research to understand (1) the quality and relevancy of the HN/HND curricula currently used by different Universities in Uganda to the national job market requirements and (2) the higher education institutional capacity readiness to offer adequate nutrition and dietetics training in Uganda is needed.

#### Limitations

Amongst the HN/HND respondents; more females than males responded to the survey. This could be attributed to the social behavioural differences in the way males and females engage in online activities; females are reported to engage more in online information exchange than males [63]. Responding to this study involved more of information exchange. Most of the graduates that responded were from Kyambogo University, this was because this University had the most graduates having started offering the program for about seven years before Makerere University.

Some of the open responses by the HN/HND graduates were quantitized and analytical judgments arrived at based on the analysis of the quantitized responses rather than the respondent’s direct narratives. The conversion process tends to strip qualitative data of its contextual meaning and may hence introduce some bias in the process [38].

Some graduates with limited access to computers and internet connections and those who perceived the study questions to be targeted to employed graduates/graduates with experience in nutrition and dietetics did not respond. The responses were hence received from a smaller group of respondents and are not be statistically representative of the entire population of HN/HND graduates that completed their undergraduate studies from the Kyambogo and Makerere Universities of Uganda in the years 2005–2016. Relatedly, responses from the employers of HN/HND graduates and organisations that host HN/HND study internships were sought and received from a purposively selected smaller number of HN/HND work/internship supervisors and may not be broadly representative of all stakeholders in this category of study participants.

## Conclusions

Based on the findings of this study, we conclude that: (1)communities of Uganda face multiple nutrition and dietetics challenges that are being addressed multisectorally; (2)HN/HND graduates trained in Uganda are well positioned to support implementation of different multisectoral nutrition and dietetics interventions but graduates possess knowledge and skills gaps even in some of the areas they are expected to exhibit competency; and (3) there is need to adequately equip HN/HND graduates trained in Uganda with the knowledge and skills required for addressng the multifaceted nutrition and dietetics challenges in the diverse national health systems settings. It is critical that the limitations in HN/HND graduates’ professional competencies as identified in this study and the weaknesses in institutional capacities to address nutrition and dietetics related challenges in different sectors of Uganda as identified in other studies [47] are addressed. Thus, the need for (1) reorienting HN/HND curricula towards the adoption of competency-based education to better equip the graduates with the knowledge and skills required for health systems performance; (2) a policy shift that encourages the development of National HN/HND training and practice standards/curriculum, the formation of formal partnerships between academic and non-academic institutions, and mainstreaming of nutrition during the legislation, policy formation and programming; and (3) undertaking research at scheduled intervals to continously evaluate the responsiveness of the HN/HND curricula to the national job requirements and the capacity of Ugandan universities to offer quality training in HN/HND  are recommended.

## Supplementary Information

**Additional file 1: Supplemental File 1**-HN-HND Graduates Consent FormR3

**Additional file 2: Supplemental File 2-**Questionnaire for HN-HND GraduatesR3

**Additional file 3: Supplemental File 3-**Questionnaire for Academic StaffsR3

**Additional file 4: Supplemental File 4-**Questionnaire for SupervisorsR3

**Additional file 5: Supplemental File 5-**Consent for Academic staffs-SupervisorsR3

## Data Availability

The questionnaires and consent forms used in this study are available as supplementary files for this paper. Due to limits required by ethical approval, the raw datasets for this study are not shareable. However additional information on the analyses undertaken can be obtained from the corresponding author on reasonable request. Contact katspk2003@gmail.com or Pkikomeko@kyu.ac.ug.

## Abbreviations

HN/HND: Human Nutrition/Human Nutrition and Dietetics
SDGs: Sustainable Development Goals
CBE: Competency-based Education
NGOs: Non-governmental organizations
URL: Uniform Resource Locator
HIV/AIDS: Human Immunodeficiency Virus/Acquired Immunodeficiency Syndrome
SPSS: Statistical Package for the Social Sciences
Ph.D.: Doctor of Philosophy
MDAs: Ministries, Departments, and Agencies

## Footnotes

1 Respectively refers to Nutrition and Dietetics professionals graduating from the Makerere University and the Kyambogo University of Uganda.

2 The system of graduation in Kyambogo and Makerere Universities of Uganda is that candidates who fulfill university academic graduation requirements either graduate towards the end of the same year in which they completed the studies, which usually is in December or in the first two months of the following year, An example is that graduates completing studies in 2016 may graduate in December of 2016 or in January or February of 2017. In this research, all candidates who indicated having graduated in 2017 actually completed studies in 2016 and hence considered as graduates of the 2016 class not the 2017 class.
