# Supplementary material for: Stakeholders’ perceptions of the nutrition and dietetics needs and the requisite professional competencies in Uganda: a cross-sectional mixed methods study
Source: BMC Health Serv Res. 2021 Jan 27;21:92. doi: 10.1186/s12913-021-06090-3 (PMC7839220; doi:10.1186/s12913-021-06090-3)
Supplement: Supplementary file 1 — Additional file 1: Supplemental File 1-HN-HND Graduates Consent FormR3 [file 12913_2021_6090_MOESM1_ESM.docx]

**Informed Consent Form for Human Nutrition/Human Nutrition and Dietetics Graduates to Participate in the Competency Needs Assessment Phase of the Study**

1. **Introduction:** Thank you for taking time to talk to me. My name is (**name of person requesting for consent**). I am one of the members on the team that is collecting data for a research study titled “Development and Validation of a Competency-Based Education Model for Strengthening Undergraduate Training of Human Nutrition and Dietetics in Uganda” This study is being undertaken as a PhD research project by Peterson Kato Kikomeko, a student at the department of Food, Nutrition and Dietetics, of the Kenyatta University Kenya and staff member of Kyambogo University. The researcher is being supervised by Dr. Sophie Ochola and Dr. Irene Ogada all from Kenyatta University and Professor Archileo Kaaya of Makerere University. Funding for undertaking this study has been received from the African Development Bank (through Kyambogo University, Uganda), Kyambogo University and the Pears Grant Run by the Hebrew University of Jerusalem, Israel. This research study has been reviewed and approved by the International Health Sciences University Ethical review board.
2. **Purpose:** The purpose of this study is to identify the competencies required of Human Nutrition/Human Nutrition and Dietetics professionals to competently perform in Uganda’s health system as well as develop and validate a Undergraduate competency-based education model suitable for use in Uganda. The objectives of the study are to:
3. To determine the population nutrition/dietetic needs in Uganda.
4. To assess the current HN/HND scope of training in Uganda.
5. To establish the competencies required of HN/HND undergraduates for health systems performance in Uganda.
6. To develop and validate a competency-based education model for the training of HN/HND at the undergraduate level in Uganda.
7. To evaluate the extent to which undergraduate HN/HND curricula currently used by academic institutions of Uganda address the competencies comprised in the developed CBE model.
8. **Study Procedures:** This research will be conducted in three phases: competency needs assessment, development of competency-based model, and validation of the competency-based model. The competency needs assessment phase will involve collecting information from different stakeholders in the field of Human Nutrition/Human Nutrition and Dietetics in Uganda including practicing Human Nutrition/Human Nutrition and Dietetics graduates, their employers (both Government and Non-government), Institutions in Uganda that offer Human Nutrition/Human Nutrition and Dietetics training at the undergraduate level, and members that sit on Sub-county Nutrition Coordination Committees in selected districts. The Model development phase will engage a few selected members representing the diversity of stakeholders engaged in the competency needs assessment phase to reflect at responses provided in the competency needs assessment phase and develop a competency framework. The Competency-based framework will highlight the key competencies agreed upon by stakeholders as being important for the training of Human Nutrition/Human Nutrition and Dietetics at the Undergraduate level in Uganda. The Model content validation phase will involve asking the different participants to rate the expected usefulness of aspects of the Competency Model developed. You may be requested to participate in one or more than one phase of the research process.
9. **Participation in the Study**: You have been identified and requested to contribute to the different phases of this study because you are a key stakeholder in health and nutrition in Uganda**.**
10. **Possible Risks of Study Participation:** This study involves very minimal possibilities for risk, stress and discomfort. There will be no collection of blood or any other biological samples from any study participant. During the competency needs assessment and content validation phases of the study, information will be collected through emailed questionnaires, in-depth interviews, key informant interviews, Phone interviews, focus group discussions, and review of documents. The questions and observations in the assessment relate to your routine daily activities in the field of Human nutrition/Human nutrition and dietetics in Uganda. Responding through either technique may take **45 minutes to one hour**.

Participation in the model development phase of the research will involve being engaged in two workshops and the Delphi technique. The workshops will each last two days and be held at a place with convenient facilities in Kampala. The Delphi technique will involve 3 rounds of soliciting responses from participants in a period of 3-6 months.

1. **Benefits and Compensations:** You will not receive any compensation for participating in the competency needs assessment phase of this study. Participants that will participate in the model development workshop will receive a modest transportation allowance to cater for transportation costs to and from the workshop venue. Depending on availability of funding, accommodation may also be provided to participants travelling from away from Kampala and Wakiso districts. This will be communicated when and invitation for participation is sent out to participants residing out of Kampala and Wakiso districts.
2. **Confidentiality**If you agree to participate in this study, your participation and any information you provide to us is completely confidential, and will only be used for purposes of this research. If you decide to participate, you are free to withdraw at any time. You will also be free not to answer certain questions, discuss certain topics, and or even put an end to the interview.
3. **Voluntary Participation**

Participation in the study is voluntary, one is free to accept or not to participate in the study, or to curtail his contribution at any step of the study process.

1. **Contacts and Questions:** If you have any questions you may contact the research supervisors Dr. Sophie Ochola on +254721449803and or Dr. Irene Oganda on +254723955466 or Prof. Archileo Kaaya on +256772440046. The researcher Peterson Kato Kikomeko can be contacted on +256 752 425 296 or email [pkikomeko@kyu.ac.ug](mailto:pkikomeko@kyu.ac.ug).

If you have questions about your rights as a participant in this study, general questions, or have complaints, concerns or issues you want to discuss with someone outside the research, call the International Health Sciences University –Research Ethical Committee Chairperson Dr. Samuel Kabwigu on (+256 779610100) & the executive secretary of UNCST on ( +256 414 -705500) respectively.

**J. Participant’s statement**

The above information regarding my participation in the study is clear to me. I have been given a chance to ask questions and my questions have been answered to my satisfaction. My participation in this study is entirely voluntary. I understand that my records will be kept private and that I can leave the study at any time**.** A copy of this consent form will be given to you if you wish. The consent form has been explained to me and I agree to take part in the study. I am free to choose for myself to be in this study, and by signing this consent form I do not waive any of my legal rights. Signing this consent form does not relieve the investigators of responsibility for adverse events as a result of my participation in this study. Signing this consent form indicates that I have been informed about the research study in which I am voluntarily agreeing to participate.

To participate in this study, please sign below:

Name of Participant ----------------------------------------------

Signature of Participant………………………………………

Date: ………………………………………………………..

Person Administering the consent form………………………………………………

Signature ………………………………………………..

Date …………………………………………………………
