# Supplementary material for: Stakeholders’ perceptions of the nutrition and dietetics needs and the requisite professional competencies in Uganda: a cross-sectional mixed methods study
Source: BMC Health Serv Res. 2021 Jan 27;21:92. doi: 10.1186/s12913-021-06090-3 (PMC7839220; doi:10.1186/s12913-021-06090-3)
Supplement: Supplementary file 2 — Additional file 2: Supplemental File 2-Questionnaire for HN-HND GraduatesR3 [file 12913_2021_6090_MOESM2_ESM.docx]

| **Questionnaire for Human Nutrition/Human Nutrition and Dietetic Professionals HN/HND in Uganda** | | | | | |
| --- | --- | --- | --- | --- | --- |
| **Date of Interview:** | | | | | |
|  | | | | | |
|  | **Background Information (Probes)** | | | Responses | |
| 001 | Gender of participant | | | 1. Male 2. Female | |
| 002 | Age (years) | | |  | |
| 003 | Training institution attended during undergraduate HN/HND training | | | 1. Kyambogo University 2. Makerere University | |
| 004 | In which organisations did you undertake HN/HND internships during undergraduate training?(Organisations and respective districts) | | |  | |
| 005 | Year graduated with undergraduate degree in HN/HND | | |  | |
| 006 | Year of first entry into professional HN/HND practice (First HN/HND job after school) | | |  | |
| 007 | Additional formal training obtained since obtaining undergraduate degree in HN/HND (e.g. post graduate studies) | | |  | |
| 008 | Current employment (Organisation, job title and respective district of employment) | | |  | |
| 009 | Organisations previously worked for in Uganda (Name of organisation, job titles, and respective districts of work) | | |  | |
| 010 | What are the job roles of your current position as defined by your organisations work contract? (Specific job roles as per work contract) | | |  | |
|  | **Assessing for Competencies Required of HN/HND Professionals in Uganda** | | | | |
|  | **Required Knowledge Amongst HN/HND Graduates for Health System Performance (Probes)** | | | |  |
| 011 | What are the different HN/HND positions available in the organisation where you are employed? (Job positions/ titles for HN/HND graduates) | | | |  |
| 012 | How many HN/HND professionals are currently employed by your organisation? (Number of HN/HND graduates employed) | | | |  |
| 013 | In what position are you currently employed by your organisation? (Current positions/job title of HN/HND graduate) | | | |  |
| 014 | What roles are you assigned/do you perform in your current work? (Job specific roles of HN/HND graduates) | | | |  |
| 015 | Which specific activities do you perform to fulfil those roles in your current work? (Specific activities done) | | | |  |
| 016 | What knowledge do HN/HND professionals need to competently perform the given roles and activities? (Knowledge required) | | | |  |
| 017 | Did you possess the required knowledge to perform the given roles upon graduation? (Underlying reasons) | | | |  |
| 018 | What strategies have enabled you to acquire your current command of the HN/HND knowledge? (Specific strategies t) | | | |  |
| 019 | What strategies would you recommend to be undertaken by HN/HND graduates to further develop the professional competency? (recommended strategies) | | | |  |
| 020 | Does your organisation undertake further on-job training for HN/HND professionals? (Number of training per year and details of last training) | | | |  |
| 021 | What knowledge does your agency focus on during on-job training of HN/HND professionals? (knowledge, skills/abilities) | | | |  |
| 022 | What knowledge does your employer expect of HN/HND graduates? (Specific HN/HND Knowledge/skills required by employer) | | | |  |
| 023 | Do you find the training you received during your HN/HND undergraduate studies correspondent to the knowledge requirements of your employers? (Underlying reasons) | | | |  |
| 024 | What gaps in nutrition knowledge have hindered you as an individual from effectively delivering nutrition/dietetic services? (Individual gaps in knowledge) | | | |  |
| 025 | What gaps in knowledge have you observed amongst fellow HN/HND professionals? (Gaps and underlying reasons) | | | |  |
| 026 | Which of the mentioned gaps in knowledge are specific to fresh HN/HND undergraduates trained in Uganda? (Knowledge gaps specific to fresh HN/HND graduates) | | | |  |
| 027 | Given your experience, what knowledge in HN/HND do you consider as relevant to attain by students of HN/HND? (Specific knowledge in HN/HND that needs to be attained during training, underlying reasons) | | | |  |
| 028 | Which of the mentioned knowledge attributes did you not attain while a student? (Specific knowledge attributes, underlying reasons) | | | |  |
| 029 | Which of the mentioned knowledge attributes have you only attained from the field? (Specific knowledge attributes attained in field? | | | |  |
| 030 | Given you current expertise, is the time allocated to the study of HN/HND appropriate to allow students gain required knowledge? (underlying reasons) | | | |  |
| 031 | What other methods would you recommend to be used in the training of HN/HND if students are to acquire the required knowledge? | | | |  |
|  | **Required Skills and Abilities Amongst HN/HND Graduates for Health System Performance (Probes)** | | | |  |
| 032 | Would you say HN/HND graduates from Uganda Universities possess adequate skills and abilities for national health system performance? (Underlying reasons) | | | |  |
| 033 | What skills/abilities do you think HN/HND graduates in Uganda should possess for health system performance? (Required skills/abilities, underlying reasons) | | | |  |
| 034 | What skills/abilities gaps in nutrition and dietetics do you have as an individual? (Individual gaps in nutrition/dietetic skills/abilities) | | | |  |
| 035 | What gaps in skills/abilities have you observed amongst fellow HN/HND professionals? (Gaps in skills/abilities among HN/HND undergraduates) | | | |  |
| 036 | What gaps in skills/abilities are specific to fresh HN/HND undergraduates trained in Uganda? (Specific gaps and underlying reasons) | | | |  |
| 037 | Given your experience, what skills/abilities do you consider as relevant to attain by students of HN/HND during undergraduate training? (Specific skills that need to be attained during training and underlying reasons) | | | |  |
| 038 | Which of the mentioned skills/abilities did you not attain while a student? (specific knowledge, skills and abilities and underlying reasons) | | | |  |
| 039 | Which of the mentioned skills/abilities have you only attained from the field? (Specific skills/ abilities attained in field? | | | |  |
| 040 | What skills does your employer expect of HN/HND graduates? (Specific HN/ skills required by employer) | | | |  |
| 041 | Do you find the training you received during your HN/HND undergraduate studies correspondent to the skills requirements of your employers? (underlying reasons) | | | |  |
| 042 | Given you current expertise, do you think the time allocated to the study of HN/HND is appropriate to allow students gain required skills/abilities? (underlying reasons) | | | |  |
| 043 | What other methods would you recommend to be used in the training of HN/HND if students are to acquire the required skills/abilities? | | | |  |
|  | **Population Nutrition Needs and Demands in Uganda** | | | | |
|  | **Addressed Population Nutrition/Dietetic Needs and Demands (Probes)** | | | |  |
| 044 | What are the nutrition and dietetic problems/challenges faced by the community/population you currently serve? (Specific nutrition and dietetic challenges) | | | |  |
| 045 | What nutrition and dietetic services does the community you serve usually demand? (Specific nutrition services demanded by the community) | | | |  |
| 046 | Which of the demanded nutrition and dietetic services does your employer provide? (Addressed community nutrition/dietetic needs. Reasons of failure to address other needs) | | | |  |
| 047 | Which other nutrition/dietetic services are provided to the community/population you serve by other agencies? (Nutrition/dietetic services by other agencies) | | | |  |
| 048 | What would you consider as the priority nutrition and dietetic services that can address the needs of the community/population served? (priority services) | | | |  |
|  | What other resources are needed to contribute to nutrition/dietetic improvements in the community you serve (Financial, physical, political, infrastructural, human) | | | |  |
| 049 | Would you say HN/HND undergraduates trained in Uganda possess adequate knowledge required to provide community/population nutrition/dietetic needs? Underlying reasons | | | |  |
| 050 | What knowledge do you think HN/HND graduates should possess for them to effectively address community/population nutrition/dietetic needs in Uganda? (Specific knowledge and underlying reasons) | | | |  |
|  | **Scope of Training and Practice of HN/HND in Uganda** | | | | |
| 051 | Are there established minimum training requirements for HN/HND in Uganda? (Examples, availability and accessibility by stakeholders. Or reasons for non-existence) | |  | | |
| 052 | Is there a need for a general national standard stipulating the minimum training requirements/expectations for HN/HND in Uganda? (Underlying reasons) | |  | | |
| 053 | | Are there national HN/HND training/practice guides/standards besides institutional HND curricula (Examples, availability and accessibility by stakeholders. Or reasons for non-existence) |  | | |
| 054 | | Do the guides reflect the required HN/HND professional competencies for performance in Uganda's Health System? (Competencies reflected) |  | | |
| 055 | Depending on your expertise, what knowledge aspects would you recommend to be considered as a minimum requirement in the training of HN/HND professionals in Uganda? (Aspects, underlying reasons) | |  | | |
| 056 | What skills/abilities aspects would you recommend to be considered as a minimum requirement in the training of HN/HND professionals in Uganda? (Skills/abilities and underlying reasons) | |  | | |
| 057 | Is the training and practice of HN/HND regulated in Uganda? ( Credentialing and accreditation agencies, underlying reasons) | |  | | |
| 058 | What other legislation(s) governs/impinges the training and practice of HN/HND in Uganda? (Examples of policy and legal provisions) | |  | | |
| 059 | What are the mandates of HN/HND professionals in Uganda as according to existent legislation? (Specific mandates of HN/HND professionals as per the legal provisions) | |  | | |
| 060 | Based on your experience, what mandates do you find appropriate for the practice of HN/HND in Uganda (Specific mandates of HN/HND professionals as per the legal provisions) | |  | | |
| 061 | Is there a defined scope of training and practice for HN/HND used by all universities in Uganda? (Aspects covered by the scope of practice) | |  | | |
| 062 | What sets of competences ought to be considered for inclusion in developing a scope of training/practice of HN/HND in Uganda? (Competences that should be reflected) | |  | | |
| 063 | What methods can be pursued to foster competency based education of HN/HND at undergraduate level in Uganda? (Methods/techniques) | |  | | |
| 064 | What are the likely challenges that may limit implementation of competency based education for HN/HND in Uganda? (Challenges that can limit CBE of HN/HND) | |  | | |
| 065 | | Do you know of any defined opportunities for continuous profession development in HN/HND graduates in Uganda? (Existing opportunities and parties responsible) |  | | |
| 066 | | What strategies can be undertaken to institute continuous profession development for HN/HND graduates in Uganda? (Specific strategies) |  | | |
| **END: Thank you so much for the Contribution** | | | | | |
