# Supplementary material for: Stakeholders’ perceptions of the nutrition and dietetics needs and the requisite professional competencies in Uganda: a cross-sectional mixed methods study
Source: BMC Health Serv Res. 2021 Jan 27;21:92. doi: 10.1186/s12913-021-06090-3 (PMC7839220; doi:10.1186/s12913-021-06090-3)
Supplement: Supplementary file 3 — Additional file 3: Supplemental File 3-Questionnaire for Academic StaffsR3 [file 12913_2021_6090_MOESM3_ESM.docx]

# **Questionnaire for Academic Staffs that Train Human Nutrition/Human Nutrition and Dietetic Professionals in Uganda**

| **Date of interview:** | | |
| --- | --- | --- |
| 001 | Gender of participant | 1. Male 2. Female |
| 002 | Name of Training institution |  |
| 003 | Position of academic staff (i.e. Asst. Lecturer, Lecturer, Dr. Professor) |  |
| 004 | Units/courses taught by respondent |  |
| 005 | Highest academic qualification of participant |  |
|  | Years of teaching experience |  |
| **Assessing for required competencies and existing competency gaps amongst HN/HND professionals** | | |
|  | **Required Knowledge Amongst HN/HND Graduates for Health System Performance (Probes)** |  |
| 006 | What are the different types of jobs/work done by HN/HND professionals in Uganda (specific jobs/domains in which HN/HND graduates are employed) |  |
| 007 | What are some of the job roles assigned/performed by HN/HND graduates |  |
| 008 | What knowledge do HN/HND professionals need to competently offer nutrition/dietetic services in Uganda? (Key knowledge attributes/areas required) |  |
| 009 | What gaps in knowledge have you observed amongst HN/HND professionals? (General gaps in knowledge amongst HN/HND graduates) |  |
| 010 | Which of the mentioned gaps in knowledge are specific to fresh HN/HND undergraduates trained in Uganda? (Knowledge gaps specific to fresh HN/HND graduates) |  |
| 011 | Given your experience, what knowledge in HN/HND do you consider as relevant to attain by students of HN/HND? (Specific knowledge in HN/HND that needs to be attained during training, underlying reasons) |  |
| 012 | Given you current expertise, is the time allocated to the study of HN/HND appropriate to allow students gain required knowledge? (underlying reasons) |  |
| 013 | What other methods would you recommend to be used in the training of HN/HND if students are to acquire the required knowledge and knowledge? |  |
| 014 | What strategies can be undertaken by HN/HND graduates to further develop their knowledge for professional competency? |  |
|  | **Required Skills and Abilities Amongst HN/HND Graduates for Health System Performance (Probes)** |  |
| 015 | Would you say HN/HND graduates in Uganda possess adequate skills and abilities for national health system performance? (Underlying reasons) |  |
| 016 | What skills/abilities gaps in nutrition and dietetics do you have as an individual? (Individual gaps in nutrition/dietetic skills/abilities) |  |
| 017 | What gaps in skills/abilities are specific to fresh HN/HND undergraduates trained in Uganda? (Specific gaps and underlying reasons) |  |
| 018 | Given your experience, what skills/abilities do you consider as relevant to attain by students of HN/HND during undergraduate training? (Specific skills that need to be attained during training and underlying reasons) |  |
| 019 | Which of the mentioned skills and abilities is currently not a focus of HN/HND training in your institution? (Knowledge, skills and abilities not learnt and underlying reasons) |  |
| 020 | Which of the mentioned skills and abilities can HN/HND graduates obtain from the field? (Skills/abilities that can be obtained in the field) |  |
|  | **Population Nutrition Needs and Demands in Uganda** | |
|  | **Addressed Population Nutrition/Dietetic Needs and Demands (Probes)** |  |
| 021 | What are the nutrition and dietetic problems/challenges faced by the community/population in Uganda? (specific nutrition and dietetic challenges) |  |
| 022 | What nutrition and dietetic services are provided to communities in Uganda? (Nutrition services provided in different communities of Uganda) |  |
| 023 | Would you say HN/HND undergraduates trained in Uganda possess adequate knowledge, skills/abilities required to provide community/population nutrition/dietetic needs? (Underlying reasons) |  |
| 024 | What knowledge should HN/HND graduates possess for them to effectively address community/population nutrition/dietetic needs in Uganda? Specific knowledge and underlying reasons |  |
| 025 | What skills/abilities should HN/HND graduates possess for them to effectively address community/population nutrition/dietetic needs in Uganda? (Specific skills/abilities and underlying reasons) |  |
|  | **Scope of Training and Practice of HN/HND in Uganda** | |
| 026 | In regards to the degree in Human Nutrition/Human Nutrition and Dietetics, what kind of curriculum is used? (Probe Traditional time based curricula versus Competency based curricula) |  |
| 027 | What would you say are the best things about the Human Nutrition/Human Nutrition and Dietetics curricula currently used by institution? |  |
| 028 | Are there any observed challenges in implementing the HN/HND curricula that is currently in use by your institution? |  |
| 029 | Are there established minimum training requirements for HN/HND in Uganda? (Examples, availability and accessibility by stakeholders. Or reasons for non-existence) |  |
| 030 | Is there a need for a general national standard stipulating the minimum training requirements for HN/HND in Uganda? (Underlying reasons) |  |
| 031 | Are there national HN/HND training/practice guides/standards besides institutional HND curricula (Examples, availability and accessibility by stakeholders. Or reasons for non-existence) |  |
| 032 | Do the guides reflect the required HN/HND professional competencies for performance in Uganda's Health System? (Competencies reflected) |  |
| 033 | Depending on your expertise, what knowledge aspects would you recommend to be considered as a minimum requirement in the training of HN/HND professionals in Uganda? (Aspects, underlying reasons) |  |
| 034 | What skills/abilities aspects would you recommend to be considered as a minimum requirement in the training of HN/HND professionals in Uganda? (Skills/abilities and underlying reasons) |  |
| 035 | Is the training and practice of HN/HND regulated in Uganda? ( Credentialing and accreditation agencies, underlying reasons) |  |
| 036 | What other legislation(s) governs/impinges the training and practice of HN/HND in Uganda? (Examples of policy and legal provisions) |  |
| 037 | What are the mandates of HN/HND professionals in Uganda as according to existent legislation? (Specific mandates per the legal provisions) |  |
| 038 | Based on your experience, what mandates do you find appropriate for the practice of HN/HND in Uganda (Specific mandates per the legal provisions) |  |
| 037 | Is there a defined scope of training and practice for HN/HND used by all universities in Uganda? (Aspects covered by the scope of practice) |  |
| 040 | What sets of competences ought to be considered for inclusion in developing a scope of training/practice of HN/HND in Uganda? (Competences reflect) |  |
| 041 | What methods can be pursued to foster competency based education of HN/HND at undergraduate level in Uganda? (Methods/techniques) |  |
| 042 | What are the likely challenges that may limit implementation of competency based education for HN/HND in Uganda? (Challenges that can limit CBE of HN/HND) |  |
| 043 | Do you know of any defined opportunities for continuous profession development in HN/HND graduates in Uganda? (Existing opportunities) |  |
| 044 | What strategies can be undertaken to institute continuous profession development for HN/HND graduates in Uganda? (Specific strategies) |  |
| **END: Thank you so much for the Contribution** | | |
