# Supplementary material for: Stakeholders’ perceptions of the nutrition and dietetics needs and the requisite professional competencies in Uganda: a cross-sectional mixed methods study
Source: BMC Health Serv Res. 2021 Jan 27;21:92. doi: 10.1186/s12913-021-06090-3 (PMC7839220; doi:10.1186/s12913-021-06090-3)
Supplement: Supplementary file 4 — Additional file 4: Supplemental File 4-Questionnaire for SupervisorsR3 [file 12913_2021_6090_MOESM4_ESM.docx]

| **Questionnaire for Employers/Supervisors of Human Nutrition/Human Nutrition and Dietetic Professionals in Uganda** | | | | | |
| --- | --- | --- | --- | --- | --- |
|  | Date of Interview | | | |  |
|  | **Background Information (Probes)** | | | Responses | |
| 001 | Gender of participant | | | 1. Male 2. Female | |
| 002 | Name of Organisation/Entity | | | |  |
| 003 | District of Operation of the Organization/Entity | | | |  |
| 004 | Position of Person Interviewed | | | |  |
| 005 | Main sections/areas of work in which HN/HND graduates are employed | | | |  |
|  | **Assessing for Competencies Required of HN/HND Professionals in Uganda** | | | | |
|  | **Required Knowledge Amongst HN/HND Graduates for Health System Performance (Probes)** | | |  | |
| 006 | What are the different HN/HND positions available in your organisation? (Job positions/ titles for HN/HND graduates) | | |  | |
| 007 | How many HN/HND professionals are currently employed by your organisation? (Number of HN/HND graduates employed) | | |  | |
| 008 | In what position are HN/HND professionals employed by your organisation? (Current positions/job title of HN/HND graduates) | | |  | |
| 009 | What roles are assigned to the different HN/HND professionals in your organisation? (Job specific roles of HN/HND graduates in different positions) | | |  | |
| 010 | Which specific activities do HN/HND professionals perform to fulfil those roles in your current work? (Specific activities done) | | |  | |
| 011 | What knowledge do HN/HND professionals need to competently perform the given roles and activities? (Knowledge required) | | |  | |
| 012 | Did the HN/HND professionals possess the required knowledge to perform the given roles on recruitment? (Underlying reasons) | | |  | |
| 017 | What strategies have enabled HN/HND professionals to acquire the current command of the HN/HND knowledge? (Specific strategies t) | | |  | |
| 018 | What strategies would you recommend to be undertaken by HN/HND graduates to further develop the professional competency? (recommended strategies) | | |  | |
| 019 | Does your organisation undertake further on-job training for HN/HND professionals? (Number of training per year and details of last training) | | |  | |
| 020 | What knowledge does your agency focus on during on-job training of HN/HND professionals? (knowledge, skills/abilities) | | |  | |
| 021 | What knowledge does your organisation expect of HN/HND graduates? (Specific HN/HND Knowledge/skills required by employer) | | |  | |
| 022 | What gaps in nutrition knowledge have you found to be hindering HN/HND professionals from effectively delivering nutrition/dietetic services? (Individual gaps in knowledge) | | |  | |
| 023 | Which of the mentioned gaps in knowledge do you find specific to fresh HN/HND undergraduates trained in Uganda? (Knowledge gaps specific to fresh HN/HND graduates) | | |  | |
| 024 | Given your experience, what knowledge in HN/HND do you consider as relevant to attain by students of HN/HND? (Specific knowledge in HN/HND that needs to be attained during training, underlying reasons) | | |  | |
| 024 | Which of the mentioned knowledge attributes can HN/HND professionals attain from the field? (Specific knowledge attributes attained in field? | | |  | |
| 025 | What other methods would you recommend for use in the training of HN/HND if students are to acquire the required knowledge? | | |  | |
|  | | **Required Skills and Abilities Amongst HN/HND Graduates for Health System Performance (Probes)** | |  | |
| 026 | | Would you say HN/HND graduates in Uganda possess adequate skills and abilities for national health system performance? (Underlying reasons) | |  | |
| 027 | | What skills/abilities do you think HN/HND graduates in Uganda should possess for health system performance? (Required skills/abilities, underlying reasons) | |  | |
| 028 | | What skills/abilities gaps in nutrition and dietetics have you observed amongst HN/HND graduates of Uganda? (Individual gaps in nutrition/dietetic skills/abilities) | |  | |
| 029 | | What gaps in skills/abilities are specific to fresh HN/HND undergraduates trained in Uganda? (Specific gaps and underlying reasons) | |  | |
| 030 | | Given your experience, what skills/abilities do you consider as relevant to attain by students of HN/HND during undergraduate training? (Specific skills that need to be attained during training and underlying reasons) | |  | |
| 031 | | Which of the mentioned skills/abilities can HN/HND graduates attain from the field? (Specific skills/ abilities attained in field? | |  | |
| 032 | | What skills does your organisation expect of HN/HND graduates? (Specific HN/ skills required by employer) | |  | |
| 033 | | Do you find the training received by HN/HND undergraduate correspondent to the skills requirements of your organisation? (underlying reasons) | |  | |
| 034 | | What other methods would you recommend to be used in the training of HN/HND if students are to acquire the required skills/abilities? | |  | |
|  | | **Population Nutrition Needs and Demands in Uganda** | | | |
|  | | **Addressed Population Nutrition/Dietetic Needs and Demands (Probes)** | |  | |
| 035 | | What are the nutrition and dietetic problems/challenges faced by the community/population you currently serve? (Specific nutrition and dietetic challenges) | |  | |
| 036 | | What nutrition and dietetic services does the community you serve usually demand? (Specific nutrition services demanded by the community) | |  | |
| 037 | | Which of the demanded nutrition and dietetic services does your organisation provide? (Addressed community nutrition/dietetic needs. Reasons of failure to address other needs) | |  | |
| 038 | | Which other nutrition/dietetic services are provided to the community/population you serve by other agencies? (Nutrition/dietetic services by other agencies) | |  | |
| 039 | | What would you consider as the priority nutrition and dietetic services that can address the needs of the community/population served? (priority services) | |  | |
| 040 | | Would you say HN/HND undergraduates trained in Uganda possess adequate knowledge required to provide community/population nutrition/dietetic needs? Underlying reasons | |  | |
| 041 | | What knowledge do you think HN/HND graduates should possess for them to effectively address community/population nutrition/dietetic needs in Uganda? (Specific knowledge and underlying reasons) | |  | |
|  | | What skills/abilities do you think HN/HND graduates should possess for them to effectively address community/population nutrition/dietetic needs in Uganda? (Specific skills/abilities and underlying reasons) | |  | |
|  | | **Scope of Training and Practice of HN/HND in Uganda** | | | |
| 043 | | Are there established minimum training requirements for HN/HND in Uganda? (Examples, availability and accessibility by stakeholders. Or reasons for non-existence) | |  | |
| 044 | | Is there a need for a general national standard stipulating the minimum training requirements/expectations for HN/HND in Uganda? (Underlying reasons) | |  | |
| 045 | | | Are there national HN/HND training/practice guides/standards besides institutional HND curricula (Examples, availability and accessibility by stakeholders. Or reasons for non-existence) |  | |
| 046 | | | Do the guides reflect the required HN/HND professional competencies for performance in Uganda's Health System? (Competencies reflected) |  | |
| 047 | | Depending on your expertise, what knowledge aspects would you recommend to be considered as a minimum requirement in the training of HN/HND professionals in Uganda? (Aspects, underlying reasons) | |  | |
| 048 | | What skills/abilities aspects would you recommend to be considered as a minimum requirement in the training of HN/HND professionals in Uganda? (Skills/abilities and underlying reasons) | |  | |
| 049 | | Is the training and practice of HN/HND regulated in Uganda? ( Credentialing and accreditation agencies, underlying reasons) | |  | |
| 050 | | What other legislation(s) governs/impinges the training and practice of HN/HND in Uganda? (Examples of policy and legal provisions) | |  | |
| 051 | | What are the mandates of HN/HND professionals in Uganda as according to existent legislation? (Specific mandates of HN/HND professionals as per the legal provisions) | |  | |
| 052 | | Based on your experience, what mandates do you find appropriate for the practice of HN/HND in Uganda (Specific mandates of HN/HND professionals as per the legal provisions) | |  | |
| 053 | | Is there a defined scope of training and practice for HN/HND used by all universities in Uganda? (Aspects covered by the scope of practice) | |  | |
| 054 | | What sets of competences ought to be considered for inclusion in developing a scope of training/practice of HN/HND in Uganda? (Competences that should be reflected) | |  | |
| 055 | | What methods can be pursued to foster competency based education of HN/HND at undergraduate level in Uganda? (Methods/techniques) | |  | |
| 056 | | What are the likely challenges that may limit implementation of competency based education for HN/HND in Uganda? (Challenges that can limit CBE of HN/HND) | |  | |
| 057 | | | Do you know of any defined opportunities for continuous profession development in HN/HND graduates in Uganda? (Existing opportunities and parties responsible) |  | |
| 058 | | | What strategies can be undertaken to institute continuous profession development for HN/HND graduates in Uganda? (Specific strategies) |  | |
| **END: Thank you so much for the Contribution** | | | | | |
